# Dynamic Response of Multilayered Polymer Functionally Graded Carbon Nanotube Reinforced Composite (FG-CNTRC) Nano-Beams in Hygro-Thermal Environment

**DOI:** 10.3390/polym13142340

**Published:** 2021-07-16

**Authors:** Rosa Penna, Giuseppe Lovisi, Luciano Feo

**Affiliations:** Department of Civil Engineering, University of Salerno, 84084 Fisciano, Italy; glovisi@unisa.it (G.L.); lfeo@unisa.it (L.F.)

**Keywords:** polymer multilayered functionally graded materials, nanocomposite, nanobeams, dynamics, local/nonlocal stress gradient elasticity, hygro-thermal loadings

## Abstract

This work studies the dynamic response of Bernoulli–Euler multilayered polymer functionally graded carbon nanotubes-reinforced composite nano-beams subjected to hygro-thermal environments. The governing equations were derived by employing Hamilton’s principle on the basis of the local/nonlocal stress gradient theory of elasticity (L/NStressG). A Wolfram language code in Mathematica was written to carry out a parametric investigation on the influence of different parameters on their dynamic response, such as the nonlocal parameter, the gradient length parameter, the mixture parameter and the hygro-thermal loadings and the total volume fraction of CNTs for different functionally graded distribution schemes. It is shown how the proposed approach is able to capture the dynamic behavior of multilayered polymer FG-CNTRC nano-beams under hygro-thermal environments.

## 1. Introduction

Polymer nanocomposites are widely used in several fields, ranging from the field of engineering at a macroscale to the nanoscience and nanotechnology fields in order to develop high performance nanodevices (nanosensors, nanoactuators and nanogears) and nanosystems (MEMS/NEMS), especially designed for harsh environments, while also managing extreme temperatures, humidity and vibration [1,2].

It is well-known how polymer nanocomposites are commonly reinforced by various types of nanofillers to improve their mechanical and physical properties due to the large interfacial area between polymers and nanofillers [3,4]. Based on their dimensions, nanofillers can be classified into the following three different types: two-dimensional (2-D), such as graphene [5,6,7]; one dimensional (1-D), such as carbon nanotubes [8]; zero dimensional (0-D), which include silica nanoparticles and ZnO quantum dots [9,10]. Several investigations have shown that the addition of small amounts of nanofillers can considerably improve the properties of polymeric composites [11]. However, many of these studies are only useful in establishing some basic aspects including processing, characterization and the stress–strain behavior of the nanocomposites [12,13].

In current applications, reinforcements based on graphene nanoplatelets (GNPs) and carbon nanotubes (CNTs) have been widely adopted in place of conventional fiber bulk due to their exceptional properties, to enhance the mechanical, electrical and thermal properties of composite structures. To develop their use in current applications, it is necessary to observe the overall response of the nanocomposite structural element.

Notwithstanding a number of studies have been carried out on the mechanical behavior of macroscopical structures like beams [14,15,16,17,18,19,20], plates and shells [21,22,23,24,25], made of functionally graded carbon nanotubes (FG-CNTRC) or graphene nanoplatelets reinforced composites (FG-GNPRC), there is relatively little scientific knowledge about their size-dependent mechanical response at small scale. The FG-CNTRC nano-beam has become a potential candidate for a wide variety of nanosystems and the size effects on their statical and dynamic response should be further developed. To the best of our knowledge, some reference works have been developed by Borjalilou et al. [26] and Daikh et al. [27] on the bending, buckling and free vibration of FG-CNTRC composite nano-beams, and by Daikh et al. [28] on the buckling analysis of CNTRC curved sandwich nano-beams in a thermal environment. So far, no analysis has been carried out on the dynamic response of multilayered FG-CNTRC nano-beams in a hygro-thermal environment.

Consequently, the main aim of this study is to examine the size-dependent linear vibration response of multilayered polymer nano-beams reinforced with carbon nanotubes (CNTs) whose properties are temperature-dependent. As it has been widely demonstrated by the experiments at a small scale [29], nanostructures exhibit size effects in their mechanical behavior that can be accurately predicted by resorting several size-dependent continuum theories of elasticity including both nonlocal theories of elasticity and nonlocal gradient ones and local–nonlocal mixture constitutive models or coupled theories based on the combination of pure nonlocal theory with the surface theory of elasticity.

These theories are able to capture different types of size effects: nonlocal theories are able to predict only the softening or hardening material response as opposed to nonlocal gradient ones that can predict both the softening and hardening behaviors of the material at a nanoscale. In the framework of nonlocal elasticity, two of the most notable purely nonlocal constitutive laws are the softening or Eringen’s strain-driven nonlocal integral model (StrainDM) [30,31], in which the total stress of a given point is a function of the strain at all the other adjacent points of the continuum, and the more recently hardening or stress-driven nonlocal integral model (StressDM) developed by Romano and Barretta [32], in which the strain at any point results from the stress of all the points. As widely discussed in [33,34], the differential formulation of StrainDM is ill-posed and leads to the unexpected paradoxical results for some boundary and loading conditions, unlike the well-posed StressDM that provides a consistent approach for the analysis of nanostructures [35,36,37,38,39,40,41,42,43,44].

In addition, Lim et al. [45] introduced the nonlocal strain gradient theory (Lim’s NStrainGT) to generalize the Eringen’s nonlocal model by combining it with the strain gradient model in which the total stress is a function of the strain and its gradient not only at the reference point, but also at all the other points within the domain.

Although this model has been extensively applied for many years by several researchers in a large number of investigations, recently Zaera et al. [46] declared that the nonlocal strain gradient theory leads to ill-posed structural problems since the constitutive boundary conditions are in conflict with both non-standard kinematic and static higher-order boundary conditions. The ill-posed problem related to the Lim’s NStrainGT model may be bypassed by resorting to the Eringen local-nonlocal mixture constitutive model [47,48,49] or by using coupled theories based on the combination of pure nonlocal theory with the surface theory of elasticity [50,51]. The ill-posedness of Lim’s NStrainGT can be advantageously circumvented using the variationally consistent nonlocal gradient formulations, such as local/nonlocal strain-driven gradient (L/NStrainG) and local/nonlocal stress-driven gradient (L/NStressG) theories, conceived by Barretta et al. in [52,53] for both the static and dynamics problems. These novel constitutive formulations lead to well-posed static [54] and dynamic problems [55] of nanomechanics.

The main aim of this work is to study the dynamic response of multilayered polymer functionally graded carbon nanotubes-reinforced composite nano-beams subjected to hygro-thermal environments by using the aforementioned novel consistent nonlocal gradient formulations [54,55].

The main assumptions and simplifications used for studying the nonlocal vibration characteristics of FG-CNTRC composite nano-beams within hygro-thermal environments are the following:

- A slender and perfectly straight nano-beam of a Euler–Bernoulli type, with a rectangular cross-section, is considered; hence, the influence of thickness stretching and shear deformation are neglected;
- The multilayered nano-beam is composed by laminae with the same thickness and made of an isotropic polymer matrix reinforced by single walled carbon nanotubes (SWCNTs);
- Three CNTs distribution schemes are considered: a uniform distribution and two different non-uniform functionally graded distributions;
- The effective material mechanical properties are obtained by a combination of Mori-Tanaka scheme with the rule of mixtures and molecular dynamics and are assumed to be temperature dependent;
- A uniform distribution for both temperature and moisture fields through the thickness is assumed to occur in the thickness direction only.

The present paper is structured as follows. The problem formulation of multilayered FG-CNTRC nano-beams with temperature-dependent properties and the equations of motion of the multilayered Bernoulli–Euler nano-beams are derived in Section 2 by using the Hamilton’s principle. In Section 3, the local/nonlocal stress-driven gradient model of elasticity is introduced. In Section 4, the equation of linear transverse free vibration is obtained, whose solution procedure is reported in Appendix A. Finally in Section 5, the main results of a linear free vibration analysis are presented and discussed. Some closing remarks are provided in Section 6.

## 2. Problem Formulation

### 2.1. Multilayered FG-CNTRC Nano-Beam with Temperature-Dependent Properties

**Figure 1:**
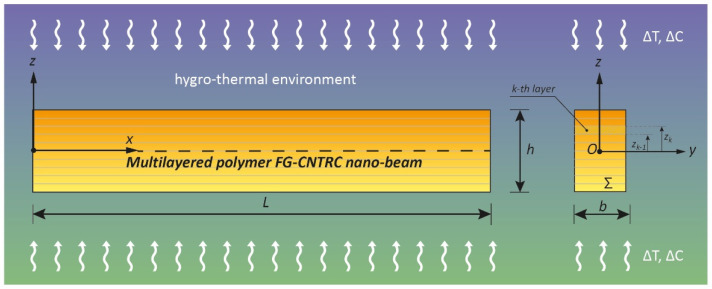
Coordinate system and configuration of a multilayered FG-CNTRC nano-beam subjected to a hygro-thermal environment.

Consider a Bernoulli–Euler multilayered FG-CNTRC nano-beam of length “*L*”, with a rectangular cross-section (*Σ*) with thickness “*h*” and width “*b*”, as illustrated in Figure 1 where the principal axes of geometric inertia, denoted by *y* and *z*, originating at the geometric center *O* of *Σ* are also shown.

It is assumed that the nano-beam is composed of ten laminae with the same thickness (*h/10*) and that each lamina (*k*) is made of an isotropic polymer matrix reinforced by SWCNTs, whose elastic properties, *P*_0_, are summarized in Table 1 as functions of the temperature and moisture distributions through the thickness, *T (z)* and *H (z)*, respectively. It is worth noting that in this work, we assumed a uniform distribution for both the temperature and moisture fields through the thickness

(1) $$T=T\left(z\right)=T_{0}+∆T=const\;$$

(2) $$H=H\left(z\right)=H_{0}+∆H=const$$

being ∆T and ∆H the temperature and moisture rises starting from initial values of the bottom surface temperature, $T_{0}=$ 300 [K] and moisture $H_{0}=0\;\left[\%wt.H_{2}O\right]$.

**Figure 2:**
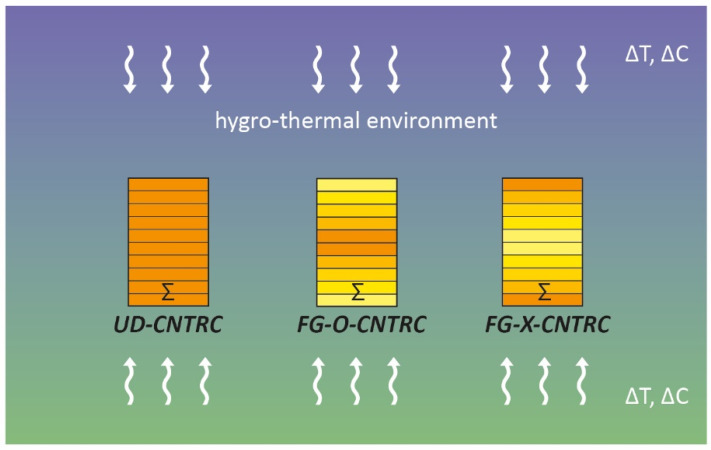
Schematic representation of a multilayered FG-CNTRC nano-beam with different patterns of CNTs dispersions: uniform distribution (UD-CNTRC); non-uniform functionally graded distributions type “O” (FG-O-CNTRC); non-uniform functionally graded distributions type “X” (FG-X-CNTRC).

In particular, three different CNTs distribution schemes have been considered: a uniform distribution, indicated as UD-CNTRC, and two non-uniform functionally graded distributions, identified as FG-O-CNTRC and FG-X-CNTRC, respectively (Figure 2).

By denoting with $\;V_{CNTs}^{\left(k\right)}\;$ the CNTs volume fraction of the *k*-th layer ($\Sigma^{\left(k\right)}$), the three selected distribution configurations can be mathematically expressed by the following relations [27]

- UD-CNTRC multilayered nano-beam
  (3) $$V_{CNTs}^{\left(k\right)}=V_{CNTs}^{*}\;$$

- FG-O-CNTRC multilayered nano-beam
  (4) $$V_{CNTs}^{\left(k\right)}=2\left(1-\frac{\left|2\right|z\left|-\left|z_{k-1}+z_{k}\right|\right|\;}{z_{k}-z_{k-1}}\right)V_{CNTs}^{*}$$

- FG-X-CNTRC multilayered nano-beam

(5) $$V_{CNTs}^{\left(k\right)}=2\frac{\left|2\right|z\left|-\left|z_{k-1}+z_{k}\right|\right|\;}{z_{k}-z_{k-1}}V_{CNTs}^{*}\;$$

where $z_{k-1}$ and $z_{k}$ refer to the vertical positions of the bottom surface and top surface of the *k*-th lamina of the multilayer nano-beam. Moreover, the symbol $V_{CNTs}^{*}$ denotes the total volume fraction of CNTs, expressed as

(6) $$V_{CNTs}^{*}=\frac{W_{CNTs}}{W_{CNTs}+\left(\frac{\rho_{CNTs}}{\rho_{m}}\right)\left(1-W_{CNTs}\right)}$$

where *W_CNTs_* is the CNTs mass fraction, while $\rho_{CNTs}$ and $\rho_{m}$ denote the CNTs mass density and the polymer matrix one, respectively.

In order to determine the effective material mechanical properties across the plane directions (*x,z*), a combination of the Mori-Tanaka scheme with the rule of mixtures and molecular dynamics is here developed, as suggested in [28]. In detail, the effective mechanical properties of the nanocomposite material, in terms of Young’s moduli $E_{11}^{\left(k\right)}\;$ (along *x*-direction) and $E_{22}^{\left(k\right)}$ (along *z*-direction) and the shear modulus, $G_{12}^{\left(k\right)}$, for the *k*-th layer are given as

(7) $$E_{11}^{\left(k\right)}=\eta_{1}V_{CNTs}^{\left(k\right)}E_{11}^{CNTs}+V_{m}^{\left(k\right)}E_{m}\;$$

(8) $$\frac{\eta_{2}}{E_{22}^{\left(k\right)}}=\frac{V_{CNTs}^{\left(k\right)}}{E_{22}^{CNTs}}+\frac{V_{m}^{\left(k\right)}}{E_{m}}$$

(9) $$\frac{\eta_{3}}{G_{12}^{\left(k\right)}}=\frac{V_{CNTs}^{\left(k\right)}}{G_{12}^{CNTs}}+\frac{V_{m}^{\left(k\right)}}{G_{m}}$$

in which $E_{11}^{CNTs}$, $E_{22}^{CNTs}$ and $\;G_{12}^{CNTs}$ are the Young’s moduli and the shear modulus of SWCNTs, respectively, and $E_{m}$ and $G_{m}\;$ are the elastic properties of the polymer matrix. In the previous equations, $V_{m}^{\left(k\right)}=1-V_{CNTs}^{\left(k\right)}$, is the polymer matrix volume fraction of *k*-th layer and $\eta_{1}$*,*$\;\eta_{2}$ $\mathrm{and}\;\eta_{3}$ are the values of the SWCNTs efficiency parameters listed in Table 2 as given in [19].

In addition, based on the rule of mixture, the Poisson’s ratio, $\nu_{12}^{\left(k\right)}$, mass density, $\rho^{\left(k\right)}$, and thermal expansion coefficient, $\alpha_{11}^{\left(k\right)}$, of the *k*-th layer are given as

(10) $$\nu_{12}^{\left(k\right)}=V_{m}^{\left(k\right)}\nu_{m}+V_{CNTs}^{\left(k\right)}\nu_{12}^{CNTs}$$

(11) $$\rho^{\left(k\right)}=V_{m}^{\left(k\right)}\rho_{m}+V_{CNTs}^{\left(k\right)}\rho_{CNTs}$$

(12) $$\alpha_{11}^{\left(k\right)}=V_{m}^{\left(k\right)}\alpha_{m}+V_{CNTs}^{\left(k\right)}\alpha_{11}^{CNTs}$$

where $\nu_{m}$ and $\nu_{12}^{CNTs}$ and $\alpha_{m}$ and $\alpha_{11}^{CNTs}$ are the Poisson’s ratios and the thermal expansion coefficients of the polymer matrix and CNTs, respectively.

Moreover, the moisture coefficient, $\beta^{\left(k\right)}$, of the nanocomposite is assumed equal to the moisture coefficient of the matrix, $\beta_{m}$, since the matrix absorbs all the water content

(13) $$\beta^{\left(k\right)}=\beta_{m}$$

### 2.2. Governing Equation

Based on Bernoulli–Euler theory, the Cartesian components of the displacement field of the multilayered FG-CNTRC nano-beam, in the elastic coordinate reference system {*O*, *x*, *y*, *z*} can be expressed by

(14) $$u_{x}\left(x,z,t\right)=u\left(x,t\right)+z\varphi_{y}\left(x,t\right)$$

(15) $$u_{y}\left(x,z,t\right)=0$$

(16) $$u_{z}\left(x,z,t\right)=w\left(x,t\right)$$

where $u_{x}\left(x,z,t\right)$, $u_{y}\left(x,z,t\right)$ and $u_{z}\left(x,z,t\right)$ are the displacement components along *x, y* and *z* directions; u(x,t) and w(x,t) are the axial and transverse displacements of the geometric center *O,* at time t, respectively; $\varphi_{y}\left(x,t\right)=-\frac{\partial w}{\partial x}\left(x,t\right)$ is the rotation of the nano-beam cross section about *y*-axis.

According to von Kármán geometrical nonlinearity, which includes small strains but moderately large rotation, the only nonzero elastic strain is given by the following relation [44]

(17) $$\varepsilon_{xx}\left(x,t\right)=\frac{\partial u\left(x,t\right)}{\partial x}-z\frac{\partial^{2}w\left(x,t\right)}{\partial x^{2}}+\frac{1}{2}{\left(\frac{\partial w}{\partial x}\left(x,t\right)\right)}^{2}$$

It is well-known how the nonlinear equations of motion can be obtained using Hamilton’s principle

(18) $$\int_{t_{1}}^{t_{2}}\left(\delta U-\delta K+\delta W\right)dt=0$$

where the expression of δK  (variation of kinetic energy), δU (variation of strain energy) and δW (variation work done by external forces) are given in the following.

The variation of kinetic energy is

(19) $$\delta K=\frac{1}{2}b\overset{L}{\underset{0}{\int }}\overset{N_{L}}{\underset{k=1}{\sum }}\overset{z_{k}}{\underset{z_{k-1}}{\int }}\rho^{\left(k\right)}{\dot{u}}_{i}\delta \dot{u}dzdx=\;\frac{1}{2}b\overset{L}{\underset{0}{\int }}\overset{N_{L}}{\underset{k=1}{\sum }}\overset{z_{k}}{\underset{z_{k-1}}{\int }}\rho^{\left(k\right)}\overset{N_{L}}{\underset{k=1}{\sum }}\left[\left(\dot{u}+z{\dot{\varphi }}_{y}\right)\left(\delta \dot{u}+z\delta {\dot{\varphi }}_{y}\right)+\dot{w}\delta \dot{w}\right]dzdx=\;\overset{L}{\underset{0}{\int }}(A_{\rho }\dot{u}\delta \dot{u}+I_{\rho }{\dot{\varphi }}_{y}\delta {\dot{\varphi }}_{y}+A_{\rho }\dot{w}\delta \dot{w})dx$$

where *k* = 1, 2, …, *N_L_* is the total number of layers of the FG nano-beam; $A_{\rho }$ and $I_{\rho }$ are the effective cross-sectional mass of the FG nano-beam and the rotary inertia of its cross-section *Σ*, respectively, expressed as

(20) $$\left\{A_{\rho },\;I_{\rho }\right\}=b\overset{N_{L}}{\underset{k=1}{\sum }}\left(\rho^{\left(k\right)}\int_{z_{k-1}}^{z_{k}}\left\{1,\;z^{2}\right\}dz\right)$$

Considering the hygro-thermal effects, the variation of strain energy of the FG nanobeams can be written as

(21) $$\delta U=\frac{1}{2}\int_{0}^{L}\int_{\Sigma }\sigma_{xx}\delta \varepsilon_{xx}d\Sigma dx=\;\frac{1}{2}\int_{0}^{L}\left(N\left(\frac{\partial \delta u\left(x,t\right)}{\partial x}+\frac{1}{2}{\left(\frac{\partial \delta w}{\partial x}\left(x,t\right)\right)}^{2}\right)-M\frac{\partial^{2}\delta w\left(x,t\right)}{\partial x^{2}}\right)dx$$

where N and M are the axial and moment stress resultants, respectively

(22) $$N=N\left(x,t\right)=A_{E}\left(\frac{\partial u\left(x,t\right)}{\partial x}+\frac{1}{2}{\left(\frac{\partial w}{\partial x}\left(x,t\right)\right)}^{2}\right)$$

(23) $$M=M\left(x,t\right)=-I_{E}\frac{\partial^{2}w\left(x,t\right)}{\partial x^{2}}$$

The stiffness components $A_{E}\;$ and $I_{E}\;$ are defined as

(24) $$\left\{A_{E},\;I_{E}\right\}=\overset{\frac{h}{2}}{\underset{-\frac{h}{2}}{\int }}Q_{11}\left\{1,\;z^{2}\right\}dz$$

being $Q_{11}$ the equivalent stiffness

(25) $$Q_{11}=\overset{N_{L}}{\underset{k=1}{\sum }}\left(\int_{z_{k-1}}^{z_{k}}Q_{11}^{\left(k\right)}dz\right)$$

which can be expressed as a function of the reduced stiffness, $Q_{11}^{\left(k\right)},\;$ of the *k*-th layer as follows

(26) $$Q_{11}^{\left(k\right)}=\frac{E_{11}^{\left(k\right)}}{1-{\left(\upsilon_{12}^{\left(k\right)}\right)}^{2}}$$

By manipulating Equations (24)–(26), the stiffness components $A_{E}\;$ and $I_{E}\;$ can be rewritten as

(27) $$\left\{A_{E},\;I_{E}\right\}=\overset{N_{L}}{\underset{k=1}{\sum }}\overset{z_{k}}{\underset{z_{k-1}}{\int }}Q_{11}^{\left(k\right)}\left\{1,\;z^{2}\right\}dz$$

Finally, the expression of the variation virtual work of the external force can be expressed by

(28) $$\delta W=-\frac{1}{2}\int_{0}^{L}(N^{T}+N^{H})\frac{\partial w\left(x,t\right)}{\partial x}\frac{\partial \delta w\left(x,t\right)}{\partial x}$$

where $N^{T}$ and $N^{H}\;$ denote the hygro-thermal axial force resultants, respectively, defined as follows

(29) $$N^{T}=N^{T}\left(z\right)=\overset{N_{L}}{\underset{k=1}{\sum }}\overset{z_{k}}{\underset{z_{k-1}}{\int }}Q_{11}^{\left(k\right)}\alpha^{\left(k\right)}∆Tdz$$

(30) $$N^{H}=N^{H}\left(z\right)=\overset{N_{L}}{\underset{k=1}{\sum }}\overset{z_{k}}{\underset{z_{k-1}}{\int }}Q_{11}^{\left(k\right)}\beta^{\left(k\right)}∆Hdz$$

By substituting Equations (19), (21) and (28) into the Hamilton’s principle, performing integration-by-parts with respect to *t* and *x* to relieve the generalized displacements δu, δw and $\delta \varphi_{y}$ of any differentiations, and using the fundamental Lemma of differential calculus, we obtain the following equations of motion

(31) $$\frac{\partial N\left(x,t\right)}{\partial x}=A_{\rho }\frac{\partial^{2}u\left(x,t\right)}{\partial t^{2}}$$

(32) $$\frac{\partial^{2}M\left(x,t\right)}{\partial x^{2}}+\frac{\partial }{\partial x}\left(N\left(x,t\right)\frac{\partial w\left(x,t\right)}{\partial x}\right)-\left(N^{T}+N^{H}\right)\frac{\partial^{2}w\left(x,t\right)}{\partial x^{2}}=A_{\rho }\frac{\partial^{2}w\left(x,t\right)}{\partial t^{2}}-I_{\rho }\frac{\partial^{4}w\left(x,t\right)}{\partial x^{2}\partial t^{2}}$$

with the corresponding boundary conditions at *x* = [0, *L*]

(33) u(x,t)   or   N(x,t)

(34) $$-\frac{\partial w\left(x,t\right)}{\partial x}\;\mathrm{or}\;\;\;M\left(x,t\right)$$

(35) $$w\left(x,t\right)\;\;\mathrm{or}\;\;\;\;V\left(x,t\right)=\frac{\partial M\left(x,t\right)}{\partial x}+N\frac{\partial w\left(x,t\right)}{\partial x}-\left(N^{T}+N^{H}\right)\frac{\partial w\left(x,t\right)}{\partial x}$$

where V(x,t)  denotes the equivalent shear force.

Substituting Equations (22) and (23) into Equations (31) and (32), the governing equations can be rewritten in terms of displacements

(36) $$A_{E}\frac{\partial^{2}u\left(x,t\right)}{\partial x^{2}}+A_{E}\frac{\partial^{2}w\left(x,t\right)}{\partial x^{2}}=A_{\rho }\frac{\partial^{2}u\left(x,t\right)}{\partial t^{2}}$$

(37) $$-I_{E}\frac{\partial^{4}w\left(x,t\right)}{\partial x^{4}}+\frac{\partial }{\partial x}\left(\left(A_{E}\frac{\partial u\left(x,t\right)}{\partial x}+\frac{1}{2}A_{E}{\left(\frac{\partial w}{\partial x}\left(x,t\right)\right)}^{2}\right)\frac{\partial w\left(x,t\right)}{\partial x}\right)-\left(N^{T}+N^{H}\right)\frac{\partial^{2}w\left(x,t\right)}{\partial x^{2}}\;=A_{\rho }\frac{\partial^{2}w\left(x,t\right)}{\partial t^{2}}-I_{\rho }\frac{\partial^{4}w\left(x,t\right)}{\partial x^{2}\;\partial t^{2}}$$

## 3. Local/Nonlocal Stress Gradient Formulation

By denoting with x  and ξ  the position vectors of the points of the domain at time *t*, with $\sigma_{xx}\;$ and $\frac{\partial \sigma_{xx}\;}{\partial_{x}}$ the axial stress component and its gradient and with $\xi_{1}$ and $L_{l}$ the mixture and the gradient length parameters, respectively, the elastic axial strain component, ${\;\varepsilon }_{xx}$, can be expressed by the well-known constitutive mixture equation (local/nonlocal stress gradient integral formulation [52])

(38) $$\varepsilon_{xx}=\xi_{1}\frac{\sigma_{xx}\left(x,t\right)}{Q_{11}}+\frac{1-\xi_{1}}{Q_{11}}\;\int_{0}^{L}\Phi_{L_{c}}\left(x-\xi \right)\sigma_{xx}\left(\xi ,t\right)d\xi -\frac{1}{Q_{11}}L_{l}^{2}\frac{\partial }{\partial x}\int_{0}^{L}\Phi_{L_{c}}\left(x-\xi \right)\frac{\partial \sigma_{xx}\left(\xi ,t\right)}{\partial x}d\xi$$

where $\Phi_{L_{c}}\;$ is the biexponential function of the scalar averaging kernel depending on the length-scale parameter, $L_{c},$ which describe the nonlocal effects.

By assuming the following smoothing function

(39) $$\Phi_{L_{c}}\left(x,\;L_{c}\right)=\frac{1}{2L_{c}}\exp\;(-\frac{\left|x\right|}{L_{c}}\;)$$

Equation (38) can be rewritten as

(40) $$\varepsilon_{xx}-L_{c}^{2}\frac{\partial^{2}\varepsilon_{xx}}{\partial x^{2}}=\frac{\sigma_{xx}}{Q_{11}}-\frac{L_{c}^{2}}{Q_{11}}\left(\xi_{1}+\frac{L_{l}^{2}}{L_{c}^{2}}\right)\frac{\;\partial^{2}\sigma_{xx}}{\partial x^{2}}$$

with the constitutive boundary conditions (CBCs) at the ends of the multilayered FG nano-beam (x=0,L)

(41) $$\frac{\partial \varepsilon_{xx}\left(0,t\right)}{\partial x}-\frac{1}{L_{c}}\varepsilon_{xx}\left(0,t\right)=-\frac{1}{Q_{11}}\frac{\xi_{1}}{L_{c}}\sigma_{xx}\left(0,t\right)+\frac{1}{Q_{11}}\left(\xi_{1}+\frac{L_{l}^{2}}{L_{c}^{2}}\right)\frac{\partial \sigma_{xx}\left(0,t\right)}{\partial x}\;$$

(42) $$\frac{\partial \varepsilon_{xx}\;\left(L,t\right)}{\partial x}+\frac{1}{L_{c}}\varepsilon_{xx}\left(L,t\right)=\frac{1}{Q_{11}}\frac{\xi_{1}}{L_{c}}\sigma_{xx}\left(L,t\right)+\frac{1}{Q_{11}}\left(\xi_{1}+\frac{L_{l}^{2}}{L_{c}^{2}}\right)\frac{\partial \sigma_{xx}\left(L,t\right)}{\partial x}\;$$

Next, by substituting Equation (17) into Equations (40)–(42), then multiplying by (1, *z*), the integration over the cross section of the multilayered FG nano-beam provides the following NStressG equations

(43) $$A_{E}\frac{\partial u\left(x,t\right)}{\partial x}+\frac{1}{2}A_{E}{\left(\frac{\partial w\left(x,t\right)}{\partial x}\right)}^{2}-A_{E}L_{c}^{2}\frac{\partial^{3}u\left(x,t\right)}{\partial x^{3}}-A_{E}L_{c}^{2}\frac{\partial^{2}}{\partial x^{2}}{\left(\frac{1}{2}\frac{\partial w\left(x,t\right)}{\partial x}\right)}^{2}\;=N^{NStressG}\left(x,t\right)-L_{c}^{2}\left(\xi_{1}+\frac{L_{l}^{2}}{L_{c}^{2}}\right)\frac{\;\partial^{2}N^{NStressG}\left(x,t\right)}{\partial x^{2}}$$

(44) $$-I_{E}\frac{\partial^{2}w\left(x,t\right)}{\partial x^{2}}+I_{E}L_{c}^{2}\frac{\partial^{4}w\left(x,t\right)}{\partial x^{4}}=M^{NStressG}\left(x,t\right)-L_{c}^{2}\left(\xi_{1}+\frac{L_{l}^{2}}{L_{c}^{2}}\right)\frac{\partial^{2}M^{NStressG}\left(x,t\right)}{\partial x^{2}}\;$$

with two pairs of CBCs

(45) $$\frac{\partial^{2}u\left(0,t\right)}{\partial x^{2}}-\frac{1}{L_{c}}\frac{\partial u\left(0,t\right)}{\partial x}=-\frac{1}{A_{E}}\frac{\xi_{1}}{L_{c}}N^{NStressG}\left(0,t\right)+\frac{1}{A_{E}}\left(\xi_{1}+\frac{L_{l}^{2}}{L_{c}^{2}}\right)\;\frac{\partial N^{NStressG}\left(0,t\right)}{\partial x}$$

(46) $$\frac{\partial^{2}u\left(L,t\right)}{\partial x^{2}}+\frac{1}{L_{c}}\frac{\partial u\left(L,t\right)}{\partial x}=\frac{1}{A_{E}}\frac{\xi_{1}}{L_{c}}N^{NStressG}\left(L,t\right)+\frac{1}{A_{E}}\left(\xi_{1}+\frac{L_{l}^{2}}{L_{c}^{2}}\right)\frac{\partial N^{NStressG}\left(L,t\right)}{\partial x}\;$$

(47) $$-\frac{\partial^{3}w}{\partial x^{3}}\left(0,t\right)+\frac{1}{L_{c}}\frac{\partial^{2}w}{\partial x^{2}}\left(0,t\right)=-\frac{1}{I_{E}}\frac{\xi_{1}}{L_{c}}M^{NStressG}\left(0,t\right)+\frac{1}{I_{E}}\left(\xi_{1}+\frac{L_{l}^{2}}{L_{c}^{2}}\right)\frac{\partial M^{NStressG}\left(0,t\right)}{\partial x}$$

(48) $$-\frac{\partial^{3}w}{\partial x^{3}}\left(L,t\right)-\frac{1}{L_{c}}\frac{\partial^{2}w}{\partial x^{2}}\left(L,t\right)=\frac{1}{I_{E}}\frac{\xi_{1}}{L_{c}}M^{NStressG}\left(L,t\right)+\frac{1}{I_{E}}\left(\xi_{1}+\frac{L_{l}^{2}}{L_{c}^{2}}\right)\frac{\partial M^{NStressG}\left(L,t\right)}{\partial x}$$

where $N^{NStressG}$ and $M^{NStressG}$ are the local/nonlocal stress gradient axial force and moment resultants, respectively. Moreover, by substituting Equations (31) and (32) into Equations (43) and (44), the local/nonlocal stress gradient axial force and moment resultants can be described explicitly in terms of displacement components as follows

(49) $$N^{NStressG}\left(x,t\right)=A_{E}(\frac{\partial u\left(x,t\right)}{\partial x}-L_{c}^{2}\frac{\partial^{3}u\left(x,t\right)}{\partial x^{3}})+A_{E}\left(\frac{1}{2}{\left(\frac{\partial w\left(x,t\right)}{\partial x}\right)}^{2}-L_{c}^{2}\frac{\partial^{2}}{\partial x^{2}}{\left(\frac{1}{2}\frac{\partial w\left(x,t\right)}{\partial x}\right)}^{2}\right)\;+L_{c}^{2}\left(\xi_{1}+\frac{L_{l}^{2}}{L_{c}^{2}}\right)\frac{\partial }{\partial x}\left(A_{\rho }\frac{\partial^{2}u\left(x,t\right)}{\partial t^{2}}\right)$$

(50) $$\begin{array}{cc} M^{NStressG}\left(x,t\right) & =-I_{E}\frac{\partial^{2}w\left(x,t\right)}{\partial x^{2}}+I_{E}L_{c}^{2}\frac{\partial^{4}w\left(x,t\right)}{\partial x^{4}}\\ & +L_{c}^{2}\left(\xi_{1}+\frac{L_{l}^{2}}{L_{c}^{2}}\right)\left(A_{\rho }\frac{\partial^{2}w\left(x,t\right)}{\partial t^{2}}-I_{\rho }\frac{\partial^{4}w\left(x,t\right)}{\partial x^{2}\partial t^{2}}+\left(N^{T}+N^{H}\right)\frac{\partial^{2}w\left(x,t\right)}{\partial x^{2}}-\frac{\partial }{\partial x}\left(N\frac{\partial w\left(x,t\right)}{\partial x}\right)\right) \end{array}$$

Finally, by manipulating Equations (49) and (50) and Equations (31) and (32), the following local/nonlocal stress gradient equations of motion are derived

(51) $$A_{E}\left(\frac{\partial^{2}u\left(x,t\right)}{\partial x^{2}}-L_{c}^{2}\frac{\partial^{4}u\left(x,t\right)}{\partial x^{4}}\right)+A_{E}\left(\frac{\partial^{2}w\left(x,t\right)}{\partial x^{2}}-L_{c}^{2}\frac{\partial^{4}w\left(x,t\right)}{\partial x^{4}}\right)+L_{c}^{2}\left(\xi_{1}+\frac{L_{l}^{2}}{L_{c}^{2}}\right)A_{\rho }\frac{\partial^{4}u\left(x,t\right)}{\partial x^{2}\partial t^{2}}=A_{\rho }\frac{\partial^{2}u\left(x,t\right)}{\partial t^{2}}$$

(52) $$\begin{array}{cc} -I_{E}\frac{\partial^{4}w\left(x,t\right)}{\partial x^{4}}+ & I_{E}L_{c}^{2}\frac{\partial^{6}w\left(x,t\right)}{\partial x^{6}}\\ & +L_{c}^{2}\left(\xi_{1}+\frac{L_{l}^{2}}{L_{c}^{2}}\right)(A_{\rho }\frac{\partial^{4}w\left(x,t\right)}{\partial x^{2}\partial t^{2}}-I_{\rho }\frac{\partial^{6}w\left(x,t\right)}{\partial x^{4}\partial t^{2}}+\left(N^{T}+N^{H}\right)\frac{\partial^{4}w\left(x,t\right)}{\partial x^{4}}\\ & -\frac{\partial^{3}}{\partial x^{3}}\left(N\frac{\partial w\left(x,t\right)}{\partial x}\right))\\ & =(A_{\rho }\frac{\partial^{2}w\left(x,t\right)}{\partial t^{2}}-I_{\rho }\;\frac{\partial^{4}w\left(x,t\right)}{\partial x^{2}\;\partial t^{2}}+\left(N^{T}+N^{H}\right)\frac{\partial^{2}w\left(x,t\right)}{\partial x^{2}}\\ & -\frac{\partial }{\partial x}\left(N\frac{\partial w\left(x,t\right)}{\partial x}\right)) \end{array}$$

equipped with the following natural boundary conditions at the ends (x=0,L)

(53) $$N^{NStressG}\left(x,t\right)=\bar{N}$$

(54) $$\frac{\partial M^{NStressG}\left(x,t\right)}{\partial x}+N^{NStressG}\left(x,t\right)\frac{\partial w\left(x,t\right)}{\partial x}-\left(N^{T}+N^{H}\right)\frac{\partial w\left(x,t\right)}{\partial x}=\bar{V}$$

(55) $$M^{NStressG}\left(x,t\right)=\bar{M}$$

being $\bar{N}$, $\bar{M}$ and $\;\bar{V}$ the assigned generalized forces acting at the nano-beam ends together and with the aforementioned CBCs at the nano-beam ends given by Equations (45)–(48).

## 4. Nonlinear Transverse Free Vibration Analysis

By neglecting the term $A_{\rho }$, from the first equation of motion, we obtain

(56) $$\begin{array}{cc} N^{NStressG}\left(x,t\right)= & A_{E}\left(\frac{\partial u\left(x,t\right)}{\partial x}-L_{c}^{2}\frac{\partial^{3}u\left(x,t\right)}{\partial x^{3}}\right)\\ & +A_{E}\left(\frac{1}{2}{\left(\frac{\partial w\left(x,t\right)}{\partial x}\right)}^{2}-L_{c}^{2}\frac{\partial^{2}}{\partial x^{2}}{\left(\frac{1}{2}\frac{\partial w\left(x,t\right)}{\partial x}\right)}^{2}\right)=\;\hat{N} \end{array}$$

being $\hat{N}$ constant.

For a multilayered FG nano-beam with immovable ends, by integrating both sides of Equation (56) over the domain [0, L] yields to the following expression

(57) $$\hat{N}=\frac{A_{E}}{L}\overset{L}{\underset{0}{\int }}\left(\frac{1}{2}{\left(\frac{\partial w\left(x,t\right)}{\partial x}\right)}^{2}-L_{c}^{2}\frac{\partial^{3}w\left(x,t\right)}{\partial x^{3}}\right)dz$$

which corresponds to the “mid-plane stretching effect”.

By substituting Equation (57) into Equation (52), it follows

(58) $$\begin{array}{cc} -I_{E}\frac{\partial^{4}w\left(x,t\right)}{\partial x^{4}} & +I_{E}L_{c}^{2}\frac{\partial^{6}w\left(x,t\right)}{\partial x^{6}}\\ & +L_{c}^{2}\left(\xi_{1}+\frac{L_{l}^{2}}{L_{c}^{2}}\right)(A_{\rho }\frac{\partial^{4}w\left(x,t\right)}{\partial x^{2}\partial t^{2}}-I_{\rho }\frac{\partial^{6}w\left(x,t\right)}{\partial x^{4}\partial t^{2}}\\ & +\left(N^{T}+N^{H}-\hat{N}\right)\frac{\partial^{4}w\left(x,t\right)}{\partial x^{4}})\\ & =\left(A_{\rho }\frac{\partial^{2}w\left(x,t\right)}{\partial t^{2}}-I_{\rho }\frac{\partial^{4}w\left(x,t\right)}{\partial x^{2}\;\partial t^{2}}+\left(N^{T}+N^{H}-\hat{N}\right)\frac{\partial^{2}w\left(x,t\right)}{\partial x^{2}}\right) \end{array}$$

Finally, manipulating Equations (57) and (58) we obtain the following equation, which describes the nonlinear transverse free oscillations

(59) $$\begin{array}{cc} -I_{E}\frac{\partial^{4}w\left(x,t\right)}{\partial x^{4}}+ & I_{E}L_{c}^{2}\frac{\partial^{6}w\left(x,t\right)}{\partial x^{6}}\\ & +L_{c}^{2}\left(\xi_{1}+\frac{L_{l}^{2}}{L_{c}^{2}}\right)(A_{\rho }\frac{\partial^{4}w\left(x,t\right)}{\partial x^{2}\partial t^{2}}-I_{\rho }\frac{\partial^{6}w\left(x,t\right)}{\partial x^{4}\partial t^{2}}\\ & +\left(N^{T}+N^{H}-\left(\frac{A_{E}}{L}\overset{L}{\underset{0}{\int }}\left(\frac{1}{2}{\left(\frac{\partial w\left(x,t\right)}{\partial x}\right)}^{2}-L_{c}^{2}\frac{\partial^{3}w\left(x,t\right)}{\partial x^{3}}\right)dz\right)\right)\frac{\partial^{4}w\left(x,t\right)}{\partial x^{4}})\\ & =(A_{\rho }\frac{\partial^{2}w\left(x,t\right)}{\partial t^{2}}-I_{\rho }\frac{\partial^{4}w\left(x,t\right)}{\partial x^{2}\;\partial t^{2}}\\ & +\left(N^{T}+N^{H}-\left(\frac{A_{E}}{L}\overset{L}{\underset{0}{\int }}\left(\frac{1}{2}{\left(\frac{\partial w\left(x,t\right)}{\partial x}\right)}^{2}-L_{c}^{2}\frac{\partial^{3}w\left(x,t\right)}{\partial x^{3}}\right)dz\right)\right)\frac{\partial^{2}w\left(x,t\right)}{\partial x^{2}}) \end{array}$$

The solution procedure of the previous equations is reported in Appendix A.

## 5. Results and Discussion

A hygro-thermal linear free vibration analysis of a simply-supported Bernoulli–Euler multilayered polymer FG-CNTRC nano-beam, based on local/nonlocal stress gradient theory of elasticity, is considered as a case study in this section.

The nano-beam has a length “*L = 10 nm*” and a rectangular cross-section (Σ) with thickness “*h = 0.1 L*” and width “*b = 0.1 L*”, whose material properties are listed in Table 1.

Firstly, we present the combined effects of the uniform temperature rise, ∆T, and the total volume fraction of CNTs, $V_{CNTs}^{*}$, on the dimensionless bending stiffness, $\bar{I_{E}}$, considering both a uniform distribution (UD CNTRC) and two non-uniform functionally graded distributions (FG-O CNTRC and FG-X CNTRC) along the thickness of the nano-beam (Figure 2). Then, we show the main results of the linear free vibration analysis in terms of the normalized fundamental flexural frequency ratio between the nonlocal fundamental frequency, $\tilde{\omega }$, and the dimensionless local natural frequency, ${\tilde{\omega }}_{loc}$, of a nano-beam made of a pure polymeric matrix.

### 5.1. Influence of Hygro-Thermal Loadings and Total Volume Fraction of CNTs on the Dimensionless Bending Stiffness

In this subsection, the effects of ∆T and $V_{CNTs}^{*}$ on the dimensionless bending stiffness, $\bar{I_{E}}=\frac{I_{E}}{I_{E_{m}}}$, defined as the ratio between the bending stiffnesses of the FG-CNTRC nano-beam, $I_{E}$, and of a pure polymeric matrix nano-beam, $I_{E_{m}}$, respectively, are presented.

**Figure 3:**
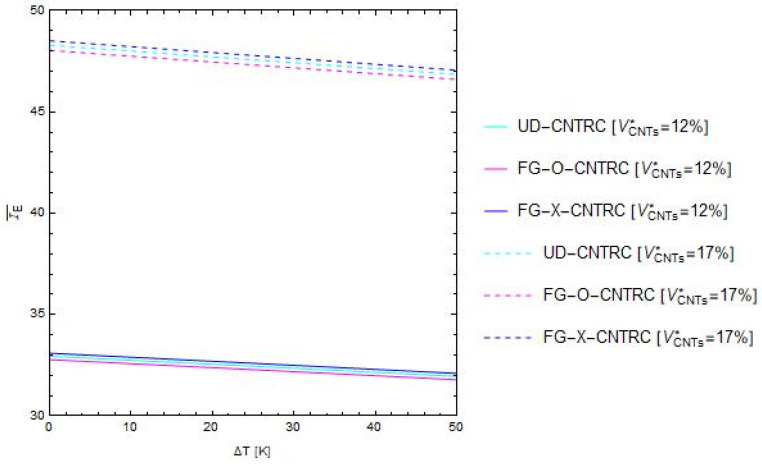
Influence of the uniform temperature rise, ∆T, on the non-dimensional bending stiffness, $\bar{I_{E}}$, varying the total volume fraction and the distribution schemes of the CNTs.

In particular, Figure 3 plots the curves of the above mentioned dimensionless bending stiffness, $\bar{I_{E}}$, versus the uniform temperature rise, ∆T*,* varying the temperature increase in the range [0, 50 (K)], the total volume fraction of CNTs in the set {12%, 17%} and considering the two non-uniform functionally graded distributions, FG-O CNTRC, FG-X CNTRC and the uniform distribution UD CNTRC, defined above (Figure 2).

Firstly, from Figure 3, it can be observed that, within the range of temperature increments here considered, the dimensionless bending stiffness decreases as ∆*T* increases. Moreover, a significant increment of the mechanical properties of the nano-beam, in terms of $\bar{I_{E}}$, is obtained as the percentage of the volume fraction of CNTs increases. Finally, it is found that the curves corresponding to the non-uniform functionally graded distribution type “X” (FG-X CNTRC) always present higher values of the dimensionless bending stiffness than those related to the case of the non-uniform functionally graded distribution type “O” (FG-O CNTRC), while the uniform distribution has an intermediate behavior (UD CNTRC).

### 5.2. Normalized Fundamental Frequency

In this subsection, the influence of hygro-thermal environment on the normalized fundamental flexural frequency of nano-beams are presented by varying both the nonlocal parameter, $\lambda_{c}$, in the range [$0.0^{+},\;0.10]$ and the gradient length parameter, $\lambda_{l}$, in the set {0.0, 0.05, 0.10}  and assuming three different values of the mixture parameter: $\xi_{1}=\left\{0.0,\;0.5,\;1.0\right\}$.

In particular, the effects of the above mentioned parameters on the behavior of a nano-beam made of pure polymeric matrix are presented in Table 3 in the case of hygro-thermal loads equal to zero, and in Table 4 in the case of uniform temperature rise and moisture concentration. Moreover, the coupled effects of the parameters $\lambda_{c}$, $\lambda_{l}$ and $\xi_{1},$ on the normalized fundamental flexural frequency of simply supported CNTRC nano-beam are summarized in the following tables:

- Table 5, Table 6 and Table 7, assuming ∆T=0 (K), $∆H=0{\;\mathrm{wt}\;\%\;H}_{2}O$, $V_{\mathrm{CNTs}}^{*}=12\%$, varying $\xi_{1}$ in the set (0.0, 0.5, 1.0), respectively;
- Table 8, Table 9 and Table 10, assuming ∆T=50 (K), $∆H=1{\;\mathrm{wt}\;\%\;H}_{2}O$, $V_{\mathrm{CNTs}}^{*}=12\%$, varying $\xi_{1}$ in the set (0.0, 0.5, 1.0), respectively;
- Table 11, Table 12 and Table 13, assuming ∆T=0 (K), $∆H=0{\;\mathrm{wt}\;\%\;H}_{2}O$, $V_{\mathrm{CNTs}}^{*}=17\%$, varying $\xi_{1}$ in the set (0.0, 0.5, 1.0), respectively;
- Table 14, Table 15 and Table 16, assuming ∆T=50 (K), $∆H=1{\;\mathrm{wt}\;\%\;H}_{2}O$, $V_{\mathrm{CNTs}}^{*}=17\%$, varying $\xi_{1}$ in the set (0.0, 0.5, 1.0), respectively.

From the numerical evidence of Table 3, Table 4, Table 5, Table 6, Table 7, Table 8, Table 9, Table 10, Table 11, Table 12, Table 13, Table 14, Table 15 and Table 16, it is interesting to note how the values of the normalized fundamental flexural frequency increased as $\lambda_{c}$ increased and decreased as the $\;\lambda_{l}$ and $\xi_{1\;}$ increased. Furthermore, as the temperature and the moisture concentration increased, the normalized fundamental flexural frequency decreased. Moreover, a hardening response was also observed when increasing the volume fraction of CNTs.

Finally, the numerical results demonstrated that the normalized fundamental flexural frequency of the FG-X CNTRC nano-beams always had greater values than those corresponding to the other distribution schemes here considered.

## 6. Conclusions

This paper considered the linear dynamic response of multilayered polymer FG carbon nanotube-reinforced Bernoulli–Euler nano-beams subjected to hygro-thermal loadings. The governing equations were derived by employing Hamilton’s principle on the basis of the local/nonlocal stress gradient theory of elasticity (L/NStressG). A Wolfram language code in Mathematica was written to carry out a parametric investigation, to check for the influence of some significant parameters on the dynamic response of a multilayered polymer FG-CNTRC simply-supported nano-beam, namely the nonlocal parameter, the gradient length parameter, the mixture parameter, the hygro-thermal loadings and the total volume fraction of CNTs for different functionally graded distribution schemes.

In view of the numerical results obtained in this paper, the main outcomes may be summarized as follows:

- A stiffening response was obtained by NStressG model when increasing the nonlocal parameter and a softening behavior was exhibited when increasing the gradient length parameter and the mixture parameter;
- Upon increasing the hygro-thermal loads it led to a decrease of the flexural frequency of the nano-beams related to a decrease in the bending stiffness due to an abatement of the thermoelastic properties of multilayered polymer FG-CNTRC nano-beams;
- By increasing the total volume fraction of CNTs, the flexural frequency of the nano-beams increased, caused by an increase in the bending stiffness; moreover, the dynamic response also depends on the functionally graded distribution schemes of CNTs.

Finally, the proposed approach was able to capture the linear dynamic response of a multilayered polymer FG-CNTRC Bernoulli–Euler nano-beam subjected to severe environmental conditions.

## Figures and Tables

**Table 1:** Thermoelastic properties of SWCNTs and the polymeric matrix.

Material	Properties	Unit	P_0_
SWCNTs	$E_{11}^{\mathrm{CNTs}}$	(GPa)	640(1−0.0005∆T)
	*ρ_cn_*	(kg/m^3^)	1350
	$\alpha_{11}^{\mathrm{CNTs}}$	(K^−1^)	$3.4584\times 10^{-6}$
	*β_cn_*	(wt. % H_2_O)^−1^	0
	$\upsilon_{11}^{\mathrm{CNTs}}$	-	0.33
Polymeric Matrix	$E_{m}$	(GPa)	3.51−0.003T+0.142H
	*ρ_m_*	(kg/m^3^)	1200
	*α_m_*	(K^−1^)	$45\left(1+0.001∆T\right)\times 10^{-6}$
	*β_m_*	(wt. % H_2_O)^−1^	0.00268
	*ν_m_*	-	0.34

**Table 2:** SWCNTs efficiency parameters.

$V_{CNTs}^{*}$	$\eta_{1}$	$\eta_{2}$	$\eta_{3}$
12 (%)	1.2833	1.0556	1.0556
17 (%)	1.3414	1.7101	1.7101

**Table 3:** Normalized fundamental flexural frequency of simply supported nano-beam of pure polymeric matrix for T=C=0.

Pure Polymeric Matrix—∆T=0 (K), $∆H=0\;\mathrm{wt}\;\%\;\;H_{2}O$									
$\lambda_{c}$	$\xi_{1}=0.0$			$\xi_{1}=0.5$			$\xi_{1}=1.0$		
	$\lambda_{l}=0.0$	$\lambda_{l}=0.05$	$\lambda_{l}=0.1$	$\lambda_{l}=0.0$	$\lambda_{l}=0.05$	$\lambda_{l}=0.1$	$\lambda_{l}=0.0$	$\lambda_{l}=0.05$	$\lambda_{l}=0.1$
0.0+	1.00000	0.98789	0.95403	1.00000	0.98789	0.95403	1.00000	0.98789	0.95403
0.01	1.00048	0.98860	0.95535	1.00024	0.98837	0.95514	1.00000	0.98814	0.95493
0.02	1.00189	0.99023	0.95756	1.00095	0.98932	0.95673	1.00000	0.98841	0.95590
0.03	1.00417	0.99272	0.96059	1.00208	0.99070	0.95876	1.00000	0.98869	0.95694
0.04	1.00724	0.99600	0.96440	1.00360	0.99248	0.96120	1.00000	0.98900	0.95804
0.05	1.01104	1.00000	0.96891	1.00548	0.99461	0.96401	1.00000	0.98931	0.95918
0.06	1.01552	1.00467	0.97408	1.00767	0.99706	0.96714	1.00000	0.98963	0.96035
0.07	1.02060	1.00993	0.97983	1.01014	0.99980	0.97056	1.00000	0.98996	0.96155
0.08	1.02623	1.01574	0.98610	1.01286	1.00277	0.97422	1.00000	0.99029	0.96275
0.09	1.03234	1.02203	0.99284	1.01579	1.00595	0.97808	1.00000	0.99061	0.96396
0.10	1.03889	1.02874	1.00000	1.01889	1.00931	0.98212	1.00000	0.99094	0.96516

**Table 4:** Normalized fundamental flexural frequency of simply supported nano-beam of pure polymeric matrix for ∆T=50 (K) and $∆H=1{\;\mathrm{wt}\;\%\;H}_{2}O$.

Pure Polymeric Matrix—∆T=50 (K), $∆H=1\;\mathrm{wt}\;\%\;\;H_{2}O$									
$\lambda_{c}$	$\xi_{1}=0.0$			$\xi_{1}=0.5$			$\xi_{1}=1.0$		
	$\lambda_{l}=0.0$	$\lambda_{l}=0.05$	$\lambda_{l}=0.1$	$\lambda_{l}=0.0$	$\lambda_{l}=0.05$	$\lambda_{l}=0.1$	$\lambda_{l}=0.0$	$\lambda_{l}=0.05$	$\lambda_{l}=0.1$
0.0+	0.81799	0.80399	0.76445	0.81799	0.80399	0.76445	0.81799	0.80399	0.76445
0.01	0.81855	0.80482	0.76600	0.81827	0.80455	0.76575	0.81799	0.80428	0.76550
0.02	0.82017	0.80671	0.76859	0.81908	0.80565	0.76762	0.81799	0.80459	0.76665
0.03	0.82279	0.80958	0.77215	0.82038	0.80725	0.77000	0.81799	0.80492	0.76787
0.04	0.82632	0.81337	0.77662	0.82214	0.80930	0.77287	0.81799	0.80527	0.76915
0.05	0.83069	0.81799	0.78190	0.82430	0.81177	0.77616	0.81799	0.80563	0.77049
0.06	0.83582	0.82336	0.78793	0.82682	0.81460	0.77982	0.81799	0.80601	0.77186
0.07	0.84164	0.82942	0.79462	0.82966	0.81775	0.78382	0.81799	0.80638	0.77326
0.08	0.84806	0.83608	0.80191	0.83278	0.82118	0.78809	0.81799	0.80676	0.77468
0.09	0.85503	0.84327	0.80972	0.83613	0.82485	0.79260	0.81799	0.80715	0.77610
0.10	0.86246	0.85093	0.81799	0.83968	0.82870	0.79729	0.81799	0.80752	0.77751

**Table 5:** Normalized fundamental flexural frequency of simply supported CNTRC nano-beam for $\xi_{1}=0.0$ and $V_{\mathrm{CNTs}}^{*}=12\%$.

$V_{CNTs}^{*}=12\%,\;$ ∆T=0 (K), $∆H=0\;\mathrm{wt}\;\%\;\;H_{2}O,\;$ $\xi_{1}=0.0$									
$\lambda_{c}$	UD CNTRC			FG-O CNTRC			FG-X CNTRC		
	$\lambda_{l}=0.0$	$\lambda_{l}=0.05$	$\lambda_{l}=0.1$	$\lambda_{l}=0.0$	$\lambda_{l}=0.05$	$\lambda_{l}=0.1$	$\lambda_{l}=0.0$	$\lambda_{l}=0.05$	$\lambda_{l}=0.1$
0.0+	5.69737	5.62835	5.43545	5.64118	5.57284	5.38184	5.75297	5.68329	5.48850
0.01	5.70012	5.63243	5.44297	5.64390	5.57688	5.38928	5.75576	5.68740	5.49609
0.02	5.70815	5.64173	5.45554	5.65186	5.58608	5.40174	5.76387	5.69679	5.50879
0.03	5.72111	5.65589	5.47285	5.66468	5.60010	5.41887	5.77694	5.71109	5.52626
0.04	5.73861	5.67456	5.49454	5.68201	5.61859	5.44034	5.79462	5.72994	5.54816
0.05	5.76030	5.69737	5.52026	5.70348	5.64118	5.46582	5.81652	5.75297	5.57414
0.06	5.78580	5.72396	5.54968	5.72873	5.66750	5.49494	5.84227	5.77982	5.60384
0.07	5.81475	5.75397	5.58243	5.75740	5.69722	5.52737	5.87150	5.81013	5.63691
0.08	5.84681	5.78705	5.61817	5.78914	5.72997	5.56276	5.90388	5.84353	5.67301
0.09	5.88164	5.82288	5.65659	5.82363	5.76545	5.60080	5.93905	5.87971	5.71180
0.10	5.91893	5.86113	5.69737	5.86055	5.80333	5.64118	5.97670	5.91834	5.75297

**Table 6:** Normalized fundamental flexural frequency of simply supported CNTRC nano-beam for $\xi_{1}=0.5$ and $V_{\mathrm{CNTs}}^{*}=12\%$.

$V_{CNTs}^{*}=12\%,\;$ ∆T=0 (K), $∆H=0\;\mathrm{wt}\;\%\;\;H_{2}O,\;$ $\xi_{1}=0.5$									
$\lambda_{c}$	UD CNTRC			FG-O CNTRC			FG-X CNTRC		
	$\lambda_{l}=0.0$	$\lambda_{l}=0.05$	$\lambda_{l}=0.1$	$\lambda_{l}=0.0$	$\lambda_{l}=0.05$	$\lambda_{l}=0.1$	$\lambda_{l}=0.0$	$\lambda_{l}=0.05$	$\lambda_{l}=0.1$
0.0+	5.69737	5.62835	5.43545	5.64118	5.57284	5.38184	5.75297	5.68329	5.48850
0.01	5.69875	5.63110	5.44177	5.64254	5.57556	5.38810	5.75437	5.68606	5.49488
0.02	5.70275	5.63651	5.45083	5.64651	5.58092	5.39707	5.75841	5.69152	5.50403
0.03	5.70920	5.64438	5.46242	5.65289	5.58871	5.40855	5.76492	5.69947	5.51574
0.04	5.71788	5.65451	5.47633	5.66148	5.59874	5.42232	5.77368	5.70970	5.52978
0.05	5.72857	5.66667	5.49232	5.67207	5.61078	5.43815	5.78448	5.72197	5.54593
0.06	5.74107	5.68064	5.51016	5.68445	5.62461	5.45582	5.79711	5.73608	5.56394
0.07	5.75516	5.69621	5.52962	5.69840	5.64003	5.47508	5.81134	5.75180	5.58359
0.08	5.77064	5.71316	5.55047	5.71373	5.65681	5.49573	5.82696	5.76892	5.60465
0.09	5.78731	5.73130	5.57250	5.73023	5.67477	5.51754	5.84380	5.78723	5.62689
0.10	5.80499	5.75043	5.59550	5.74774	5.69371	5.54031	5.86165	5.80655	5.65011

**Table 7:** Normalized fundamental flexural frequency of simply supported CNTRC nano-beam for $\xi_{1}=1.0$ and $V_{\mathrm{CNTs}}^{*}=12\%$.

$V_{CNTs}^{*}=12\%,\;$ ∆T=0 (K), $∆H=0\;\mathrm{wt}\;\%\;\;H_{2}O,\;$ $\xi_{1}=1.0$									
$\lambda_{c}$	UD CNTRC			FG-O CNTRC			FG-X CNTRC		
	$\lambda_{l}=0.0$	$\lambda_{l}=0.05$	$\lambda_{l}=0.1$	$\lambda_{l}=0.0$	$\lambda_{l}=0.05$	$\lambda_{l}=0.1$	$\lambda_{l}=0.0$	$\lambda_{l}=0.05$	$\lambda_{l}=0.1$
0.0+	5.69737	5.62835	5.43545	5.64118	5.57284	5.38184	5.75297	5.68329	5.48850
0.01	5.69737	5.62977	5.44057	5.64118	5.57425	5.38691	5.75297	5.68472	5.49367
0.02	5.69737	5.63131	5.44612	5.64118	5.57577	5.39241	5.75297	5.68627	5.49928
0.03	5.69737	5.63295	5.45206	5.64118	5.57739	5.39828	5.75297	5.68793	5.50527
0.04	5.69737	5.63467	5.45830	5.64118	5.57910	5.40447	5.75297	5.68967	5.51157
0.05	5.69737	5.63646	5.46480	5.64118	5.58086	5.41090	5.75297	5.69147	5.51813
0.06	5.69737	5.63829	5.47148	5.64118	5.58268	5.41751	5.75297	5.69332	5.52488
0.07	5.69737	5.64015	5.47829	5.64118	5.58452	5.42425	5.75297	5.69520	5.53175
0.08	5.69737	5.64202	5.48516	5.64118	5.58637	5.43106	5.75297	5.69709	5.53869
0.09	5.69737	5.64389	5.49205	5.64118	5.58823	5.43788	5.75297	5.69898	5.54565
0.10	5.69737	5.64575	5.49890	5.64118	5.59006	5.44466	5.75297	5.70085	5.55257

**Table 8:** Normalized fundamental flexural frequency of simply supported CNTRC nano-beam for $\xi_{1}=0.0$ and $V_{\mathrm{CNTs}}^{*}=17\%$.

$V_{CNTs}^{*}=17\%,\;$ ∆T=0 (K), $∆H=0\;\mathrm{wt}\;\%\;\;H_{2}O,\;$ $\xi_{1}=0.0$									
$\lambda_{c}$	UD CNTRC			FG-O CNTRC			FG-X CNTRC		
	$\lambda_{l}=0.0$	$\lambda_{l}=0.05$	$\lambda_{l}=0.1$	$\lambda_{l}=0.0$	$\lambda_{l}=0.05$	$\lambda_{l}=0.1$	$\lambda_{l}=0.0$	$\lambda_{l}=0.05$	$\lambda_{l}=0.1$
0.0+	6.87691	6.79360	6.56076	6.78835	6.70612	6.47628	6.96509	6.88072	6.64489
0.01	6.88023	6.79853	6.56984	6.79163	6.71098	6.48524	6.96846	6.88571	6.65409
0.02	6.88993	6.80975	6.58502	6.80120	6.72206	6.50022	6.97828	6.89707	6.66946
0.03	6.90556	6.82684	6.60590	6.81663	6.73893	6.52084	6.99411	6.91438	6.69061
0.04	6.92668	6.84937	6.63208	6.83749	6.76117	6.54668	7.01551	6.93721	6.71713
0.05	6.95286	6.87691	6.66314	6.86333	6.78835	6.57733	7.04202	6.96509	6.74858
0.06	6.98364	6.90900	6.69864	6.89371	6.82003	6.61238	7.07320	6.99760	6.78454
0.07	7.01859	6.94522	6.73817	6.92821	6.85579	6.65140	7.10860	7.03429	6.82457
0.08	7.05729	6.98516	6.78131	6.96641	6.89521	6.69399	7.14779	7.07473	6.86827
0.09	7.09933	7.02840	6.82768	7.00791	6.93789	6.73976	7.19037	7.11853	6.91524
0.10	7.14434	7.07458	6.87691	7.05234	6.98348	6.78835	7.23596	7.16530	6.96509

**Table 9:** Normalized fundamental flexural frequency of simply supported CNTRC nano-beam for $\xi_{1}=0.5$ and $V_{\mathrm{CNTs}}^{*}=17\%$.

$V_{CNTs}^{*}=17\%,\;$ ∆T=0 (K), $∆H=0\;\mathrm{wt}\;\%\;\;H_{2}O,\;$ $\xi_{1}=0.5$									
$\lambda_{c}$	UD CNTRC			FG-O CNTRC			FG-X CNTRC		
	$\lambda_{l}=0.0$	$\lambda_{l}=0.05$	$\lambda_{l}=0.1$	$\lambda_{l}=0.0$	$\lambda_{l}=0.05$	$\lambda_{l}=0.1$	$\lambda_{l}=0.0$	$\lambda_{l}=0.05$	$\lambda_{l}=0.1$
0.0+	6.87691	6.79360	6.56076	6.78835	6.70612	6.47628	6.96509	6.88072	6.64489
0.01	6.87857	6.79692	6.56839	6.78999	6.70940	6.48381	6.96678	6.88408	6.65262
0.02	6.88341	6.80345	6.57932	6.79477	6.71584	6.49460	6.97168	6.89070	6.66369
0.03	6.89119	6.81295	6.59332	6.80245	6.72522	6.50842	6.97956	6.90032	6.67787
0.04	6.90166	6.82517	6.61010	6.81279	6.73729	6.52499	6.99016	6.91270	6.69487
0.05	6.91457	6.83985	6.62941	6.82553	6.75177	6.54404	7.00324	6.92756	6.71442
0.06	6.92966	6.85671	6.65094	6.84043	6.76842	6.56530	7.01852	6.94464	6.73623
0.07	6.94667	6.87550	6.67443	6.85722	6.78697	6.58848	7.03575	6.96367	6.76002
0.08	6.96535	6.89597	6.69960	6.87566	6.80717	6.61333	7.05467	6.98440	6.78551
0.09	6.98547	6.91786	6.72619	6.89552	6.82877	6.63957	7.07505	7.00657	6.81244
0.10	7.00681	6.94095	6.75395	6.91658	6.85157	6.66698	7.09666	7.02996	6.84056

**Table 10:** Normalized fundamental flexural frequency of simply supported CNTRC nano-beam for $\xi_{1}=1.0$ and $V_{\mathrm{CNTs}}^{*}=17\%$.

$V_{CNTs}^{*}=17\%,\;$ ∆T=0 (K), $∆H=0\;\mathrm{wt}\;\%\;\;H_{2}O,\;$ $\xi_{1}=1.0$									
$\lambda_{c}$	UD CNTRC			FG-O CNTRC			FG-X CNTRC		
	$\lambda_{l}=0.0$	$\lambda_{l}=0.05$	$\lambda_{l}=0.1$	$\lambda_{l}=0.0$	$\lambda_{l}=0.05$	$\lambda_{l}=0.1$	$\lambda_{l}=0.0$	$\lambda_{l}=0.05$	$\lambda_{l}=0.1$
0.0+	6.87691	6.79360	6.56076	6.78835	6.70612	6.47628	6.96509	6.88072	6.64489
0.01	6.87691	6.79532	6.56694	6.78835	6.70781	6.48238	6.96509	6.88246	6.65115
0.02	6.87691	6.79717	6.57365	6.78835	6.70965	6.48900	6.96509	6.88434	6.65794
0.03	6.87691	6.79915	6.58081	6.78835	6.71160	6.49606	6.96509	6.88634	6.66519
0.04	6.87691	6.80123	6.58834	6.78835	6.71365	6.50351	6.96509	6.88844	6.67283
0.05	6.87691	6.80338	6.59618	6.78835	6.71578	6.51124	6.96509	6.89063	6.68077
0.06	6.87691	6.80559	6.60425	6.78835	6.71796	6.51921	6.96509	6.89287	6.68894
0.07	6.87691	6.80784	6.61247	6.78835	6.72017	6.52732	6.96509	6.89514	6.69726
0.08	6.87691	6.81010	6.62076	6.78835	6.72241	6.53551	6.96509	6.89743	6.70566
0.09	6.87691	6.81236	6.62908	6.78835	6.72463	6.54371	6.96509	6.89972	6.71408
0.10	6.87691	6.81460	6.63735	6.78835	6.72685	6.55188	6.96509	6.90199	6.72246

**Table 11:** Normalized fundamental flexural frequency of simply supported CNTRC nano-beam for $\xi_{1}=0.0$. $V_{\mathrm{CNTs}}^{*}=12\%$.

$V_{CNTs}^{*}=12\%,\;$ ∆T=50 (K), $∆H=1\;\mathrm{wt}\;\%\;\;H_{2}O,\;$ $\xi_{1}=0.0$									
$\lambda_{c}$	UD CNTRC			FG-O CNTRC			FG-X CNTRC		
	$\lambda_{l}=0.0$	$\lambda_{l}=0.05$	$\lambda_{l}=0.1$	$\lambda_{l}=0.0$	$\lambda_{l}=0.05$	$\lambda_{l}=0.1$	$\lambda_{l}=0.0$	$\lambda_{l}=0.05$	$\lambda_{l}=0.1$
0.0+	4.83132	4.75222	4.52921	4.81299	4.73515	4.51579	4.91626	4.83701	4.61369
0.01	4.83447	4.75690	4.53792	4.81623	4.73990	4.52450	4.91942	4.84170	4.62242
0.02	4.84366	4.76757	4.55252	4.82527	4.75040	4.53886	4.92862	4.85239	4.63703
0.03	4.85845	4.78382	4.57261	4.83983	4.76639	4.55861	4.94344	4.86867	4.65714
0.04	4.87843	4.80522	4.59776	4.85949	4.78744	4.58335	4.96346	4.89010	4.68233
0.05	4.90315	4.83132	4.62756	4.88383	4.81313	4.61266	4.98824	4.91626	4.71216
0.06	4.93218	4.86171	4.66157	4.91240	4.84304	4.64611	5.01733	4.94671	4.74622
0.07	4.96508	4.89594	4.69936	4.94479	4.87673	4.68328	5.05031	4.98101	4.78406
0.08	5.00144	4.93360	4.74051	4.98058	4.91380	4.72377	5.08676	5.01876	4.82528
0.09	5.04086	4.97430	4.78462	5.01940	4.95386	4.76717	5.12629	5.05955	4.86947
0.10	5.08298	5.01765	4.83132	5.06087	4.99655	4.81313	5.16852	5.10302	4.91626

**Table 12:** Normalized fundamental flexural frequency of simply supported CNTRC nano-beam for $\xi_{1}=0.5$. $V_{\mathrm{CNTs}}^{*}=12\%$.

$V_{CNTs}^{*}=12\%,\;$ ∆T=50 (K), $∆H=1\;\mathrm{wt}\;\%\;\;H_{2}O,\;$ $\xi_{1}=0.5$									
$\lambda_{c}$	UD CNTRC			FG-O CNTRC			FG-X CNTRC		
	$\lambda_{l}=0.0$	$\lambda_{l}=0.05$	$\lambda_{l}=0.1$	$\lambda_{l}=0.0$	$\lambda_{l}=0.05$	$\lambda_{l}=0.1$	$\lambda_{l}=0.0$	$\lambda_{l}=0.05$	$\lambda_{l}=0.1$
0.0+	4.83132	4.75222	4.52921	4.81299	4.73515	4.51579	4.91626	4.83701	4.61369
0.01	4.83290	4.75538	4.53653	4.81468	4.73840	4.52313	4.91784	4.84017	4.62102
0.02	4.83748	4.76159	4.54704	4.81919	4.74451	4.53346	4.92243	4.84639	4.63154
0.03	4.84485	4.77062	4.56049	4.82645	4.75340	4.54670	4.92981	4.85544	4.64501
0.04	4.85477	4.78224	4.57663	4.83620	4.76483	4.56257	4.93975	4.86708	4.66117
0.05	4.86698	4.79618	4.59518	4.84822	4.77854	4.58081	4.95199	4.88104	4.67974
0.06	4.88124	4.81218	4.61586	4.86226	4.79429	4.60115	4.96628	4.89708	4.70044
0.07	4.89730	4.83000	4.63838	4.87807	4.81183	4.62330	4.98238	4.91493	4.72299
0.08	4.91493	4.84938	4.66248	4.89542	4.83090	4.64701	5.00005	4.93435	4.74713
0.09	4.93390	4.87008	4.68791	4.91409	4.85128	4.67202	5.01906	4.95510	4.77260
0.10	4.95399	4.89190	4.71442	4.93387	4.87276	4.69810	5.03920	4.97697	4.79915

**Table 13:** Normalized fundamental flexural frequency of simply supported CNTRC nano-beam for $\xi_{1}=1.0$. $V_{\mathrm{CNTs}}^{*}=12\%$.

$V_{CNTs}^{*}=12\%,\;$ ∆T=50 (K), $∆H=1\;\mathrm{wt}\;\%\;\;H_{2}O,\;$ $\xi_{1}=1.0$									
$\lambda_{c}$	UD CNTRC			FG-O CNTRC			FG-X CNTRC		
	$\lambda_{l}=0.0$	$\lambda_{l}=0.05$	$\lambda_{l}=0.1$	$\lambda_{l}=0.0$	$\lambda_{l}=0.05$	$\lambda_{l}=0.1$	$\lambda_{l}=0.0$	$\lambda_{l}=0.05$	$\lambda_{l}=0.1$
0.0+	4.83132	4.75222	4.52921	4.81299	4.73515	4.51579	4.91626	4.83701	4.61369
0.01	4.83132	4.75385	4.53513	4.81313	4.73690	4.52176	4.91626	4.83864	4.61962
0.02	4.83132	4.75562	4.54156	4.81313	4.73863	4.52808	4.91626	4.84041	4.62606
0.03	4.83132	4.75750	4.54844	4.81313	4.74048	4.53485	4.91626	4.84229	4.63295
0.04	4.83132	4.75947	4.55568	4.81313	4.74243	4.54197	4.91626	4.84427	4.64020
0.05	4.83132	4.76152	4.56322	4.81313	4.74444	4.54938	4.91626	4.84632	4.64774
0.06	4.83132	4.76362	4.57097	4.81313	4.74651	4.55701	4.91626	4.84843	4.65551
0.07	4.83132	4.76576	4.57887	4.81313	4.74861	4.56477	4.91626	4.85057	4.66342
0.08	4.83132	4.76791	4.58685	4.81313	4.75073	4.57262	4.91626	4.85272	4.67140
0.09	4.83132	4.77005	4.59484	4.81313	4.75284	4.58048	4.91626	4.85487	4.67940
0.10	4.83132	4.77218	4.60279	4.81313	4.75494	4.58829	4.91626	4.85701	4.68736

**Table 14:** Normalized fundamental flexural frequency of simply supported CNTRC nano-beam for $\xi_{1}=0.0$. $V_{\mathrm{CNTs}}^{*}=17\%$.

$V_{CNTs}^{*}=17\%,\;$ ∆T=50 (K), $∆H=1\;\mathrm{wt}\;\%\;\;H_{2}O,\;$ $\xi_{1}=0.0$									
$\lambda_{c}$	UD CNTRC			FG-O CNTRC			FG-X CNTRC		
	$\lambda_{l}=0.0$	$\lambda_{l}=0.05$	$\lambda_{l}=0.1$	$\lambda_{l}=0.0$	$\lambda_{l}=0.05$	$\lambda_{l}=0.1$	$\lambda_{l}=0.0$	$\lambda_{l}=0.05$	$\lambda_{l}=0.1$
0.0+	5.88447	5.78984	5.52320	5.86092	5.76836	5.50769	6.02236	5.92752	5.66049
0.01	5.88824	5.79544	5.53362	5.86461	5.77383	5.51787	6.02614	5.93313	5.67092
0.02	5.89923	5.80820	5.55107	5.87536	5.78632	5.53492	6.03715	5.94592	5.68839
0.03	5.91693	5.82764	5.57507	5.89268	5.80533	5.55839	6.05489	5.96540	5.71242
0.04	5.94084	5.85323	5.60515	5.91607	5.83036	5.58778	6.07886	5.99105	5.74253
0.05	5.97042	5.88447	5.64077	5.94502	5.86092	5.62260	6.10852	6.02236	5.77820
0.06	6.00516	5.92083	5.68143	5.97902	5.89649	5.66235	6.14336	6.05880	5.81892
0.07	6.04455	5.96179	5.72661	6.01757	5.93658	5.70653	6.18285	6.09987	5.86418
0.08	6.08808	6.00687	5.77583	6.06018	5.98069	5.75465	6.22651	6.14507	5.91348
0.09	6.13528	6.05558	5.82860	6.10639	6.02837	5.80626	6.27386	6.19392	5.96636
0.10	6.18571	6.10749	5.88447	6.15577	6.07919	5.86092	6.32446	6.24599	6.02236

**Table 15:** Normalized fundamental flexural frequency of simply supported CNTRC nano-beam for $\xi_{1}=0.5$. $V_{\mathrm{CNTs}}^{*}=17\%$.

$V_{CNTs}^{*}=17\%,\;$ ∆T=50 (K), $∆H=1\;\mathrm{wt}\;\%\;\;H_{2}O,\;$ $\xi_{1}=0.5$									
$\lambda_{c}$	UD CNTRC			FG-O CNTRC			FG-X CNTRC		
	$\lambda_{l}=0.0$	$\lambda_{l}=0.05$	$\lambda_{l}=0.1$	$\lambda_{l}=0.0$	$\lambda_{l}=0.05$	$\lambda_{l}=0.1$	$\lambda_{l}=0.0$	$\lambda_{l}=0.05$	$\lambda_{l}=0.1$
0.0+	5.88447	5.78984	5.52320	5.86092	5.76836	5.50769	6.02236	5.92752	5.66049
0.01	5.88636	5.79361	5.53195	5.86277	5.77205	5.51624	6.02425	5.93130	5.66925
0.02	5.89184	5.80104	5.54451	5.86813	5.77931	5.52852	6.02974	5.93875	5.68183
0.03	5.90066	5.81185	5.56060	5.87676	5.78988	5.54424	6.03858	5.94958	5.69793
0.04	5.91252	5.82575	5.57989	5.88836	5.80347	5.56309	6.05047	5.96350	5.71724
0.05	5.92713	5.84242	5.60206	5.90266	5.81979	5.58476	6.06512	5.98021	5.73944
0.06	5.94420	5.86157	5.62677	5.91936	5.83852	5.60892	6.08223	5.99940	5.76419
0.07	5.96343	5.88288	5.65370	5.93818	5.85937	5.63524	6.10151	6.02076	5.79115
0.08	5.98452	5.90607	5.68252	5.95882	5.88205	5.66342	6.12266	6.04401	5.82002
0.09	6.00722	5.93085	5.71293	5.98104	5.90630	5.69314	6.14542	6.06885	5.85047
0.10	6.03127	5.95696	5.74463	6.00458	5.93185	5.72414	6.16954	6.09503	5.88223

**Table 16:** Normalized fundamental flexural frequency of simply supported CNTRC nano-beam for $\xi_{1}=1.0$. $V_{\mathrm{CNTs}}^{*}=17\%$.

$V_{CNTs}^{*}=17\%,\;$ ∆T=50 (K), $∆H=1\;\mathrm{wt}\;\%\;\;H_{2}O,\;$ $\xi_{1}=1.0$									
$\lambda_{c}$	UD CNTRC			FG-O CNTRC			FG-X CNTRC		
	$\lambda_{l}=0.0$	$\lambda_{l}=0.05$	$\lambda_{l}=0.1$	$\lambda_{l}=0.0$	$\lambda_{l}=0.05$	$\lambda_{l}=0.1$	$\lambda_{l}=0.0$	$\lambda_{l}=0.05$	$\lambda_{l}=0.1$
0.0+	5.88447	5.78984	5.52320	5.86092	5.76836	5.50769	6.02236	5.92752	5.66049
0.01	5.88447	5.79179	5.53028	5.86092	5.77026	5.51461	6.02236	5.92947	5.66758
0.02	5.88447	5.79390	5.53797	5.86092	5.77233	5.52213	6.02236	5.93159	5.67528
0.03	5.88447	5.79615	5.54619	5.86092	5.77453	5.53016	6.02236	5.93384	5.68351
0.04	5.88447	5.79851	5.55485	5.86092	5.77684	5.53862	6.02236	5.93621	5.69218
0.05	5.88447	5.80096	5.56386	5.86092	5.77923	5.54742	6.02236	5.93867	5.70120
0.06	5.88447	5.80348	5.57313	5.86092	5.78169	5.55648	6.02236	5.94119	5.71047
0.07	5.88447	5.80603	5.58257	5.86092	5.78419	5.56571	6.02236	5.94374	5.71993
0.08	5.88447	5.80860	5.59210	5.86092	5.78671	5.57503	6.02236	5.94632	5.72947
0.09	5.88447	5.81117	5.60165	5.86092	5.78922	5.58436	6.02236	5.94890	5.73903
0.10	5.88447	5.81372	5.61115	5.86092	5.79171	5.59365	6.02236	5.95145	5.74855

## Appendix A. Solution Procedure

By introducing the following dimensionless quantities

$$\frac{x}{L}=\tilde{x}$$

$$\frac{w\left(x,t\right)}{L}=\tilde{w}\left(\tilde{x},t\right)$$

$$\frac{L_{c}}{L}=\lambda_{c}$$

$$\frac{L_{l}}{L}=\lambda_{l}$$

$$\frac{N^{T}}{I_{E}}L^{2}=\tilde{N^{T}}$$

$$\frac{N^{H}}{I_{E}}L^{2}=\tilde{N^{H}}$$

$$\frac{A_{\rho }L^{4}}{I_{E}}=\tilde{A_{\rho }}$$

$$\frac{1}{L^{2}}\frac{I_{\rho }}{\tilde{A_{\rho }}}=\tilde{g^{2}}$$

$$L^{2}\frac{A_{E}}{I_{E}}=\tilde{r^{2}}$$

(A1) $$\omega^{2}\tilde{A_{\rho }}={\tilde{\omega }}^{2}$$

the dimensionless governing equations of the nonlinear transverse free vibrations associated with NStressG constitutive formulation Equation (59) can be obtained as follows

(A2) $$\begin{array}{cc} \lambda_{c}^{2}\frac{\partial^{6}\tilde{w}\left(\tilde{x},t\right)}{\partial {\tilde{x}}^{6}}- & \frac{\partial^{4}\tilde{w}\left(\tilde{x},t\right)}{\partial {\tilde{x}}^{4}}\\ & +\lambda_{c}^{2}\left(\xi_{1}+\frac{\lambda_{l}^{2}}{\lambda_{c}^{2}}\right)(\tilde{N^{T}}+\tilde{N^{H}}\\ & -\left(\tilde{r^{2}}\int_{0}^{1}\left(\frac{1}{2}{\left(\frac{\partial \tilde{w}\left(\tilde{x},t\right)}{\partial \tilde{x}}\right)}^{2}-\lambda_{c}^{2}\frac{\partial^{3}\tilde{w}\left(\tilde{x},t\right)}{\partial {\tilde{x}}^{3}}\right)dz\right))\frac{\partial^{4}\tilde{w}\left(\tilde{x},t\right)}{\partial {\tilde{x}}^{4}}\\ & -(\tilde{N^{T}}+\tilde{N^{H}}-\left(\tilde{r^{2}}\int_{0}^{1}\left(\frac{1}{2}{\left(\frac{\partial \tilde{w}\left(\tilde{x},t\right)}{\partial \tilde{x}}\right)}^{2}-\lambda_{c}^{2}\frac{\partial^{3}\tilde{w}\left(\tilde{x},t\right)}{\partial {\tilde{x}}^{3}}\right)dz\right))\frac{\partial^{2}\tilde{w}\left(\tilde{x},t\right)}{{\tilde{x}}^{2}}\\ & =\tilde{A_{\rho }}\left(\frac{\partial^{2}\tilde{w}\left(\tilde{x},t\right)}{\partial t^{2}}-\tilde{g^{2}}\frac{\partial^{4}\tilde{w}\left(\tilde{x},t\right)}{\partial {\tilde{x}}^{2}\partial t^{2}}\right)\\ & -{\tilde{\omega }}^{2}\lambda_{c}^{2}\left(\xi_{1}+\frac{\lambda_{l}^{2}}{\lambda_{c}^{2}}\right)\left(\frac{\partial^{4}\tilde{w}\left(\tilde{x},t\right)}{\partial {\tilde{x}}^{2}\partial t^{2}}-\tilde{g^{2}}\frac{\partial^{6}\tilde{w}\left(\tilde{x},t\right)}{\partial {\tilde{x}}^{4}\partial t^{2}}\right) \end{array}$$

equipped with the following dimensionless standard boundary conditions and constitutive boundary conditions

(A3) $$\;\tilde{w}\left(\tilde{x},t\right)=\tilde{w^{*}}\;\mathrm{or}\;\frac{\partial {\tilde{M}}^{NStressG}\left(\tilde{x},t\right)}{\partial \tilde{x}}+\left(\tilde{r^{2}}\int_{0}^{1}\left(\frac{1}{2}{\left(\frac{\partial \tilde{w}\left(\tilde{x},t\right)}{\partial \tilde{x}}\right)}^{2}-\lambda_{c}^{2}\frac{\partial^{3}\tilde{w}\left(\tilde{x},t\right)}{\partial {\tilde{x}}^{3}}\right)dz\right)\frac{\partial w\left(x,t\right)}{\partial x}\;\;\;\;-\left(\tilde{N^{T}}+\tilde{N^{H}}\right)\frac{\partial \tilde{w}\left(\tilde{x},t\right)}{\partial \tilde{x}}=\tilde{V}$$

(A4) $$-\frac{\partial \tilde{w}\left(\tilde{x},t\right)}{\partial \tilde{x}}=\frac{\partial {\tilde{w}}^{*}}{\partial \tilde{x}}\;\mathrm{or}\;{\tilde{M}}^{NStressG}\left(\tilde{x},t\right)=\tilde{M}$$

(A5) $$-\frac{\partial^{3}\tilde{w}\left(0,t\right)}{\partial {\tilde{x}}^{3}}+\frac{1}{\lambda_{c}}\frac{\partial^{2}\tilde{w}\left(0,t\right)}{\partial {\tilde{x}}^{2}}=-\frac{\xi_{1}}{\lambda_{c}}{\tilde{M}}^{NStressG}\left(0,t\right)+\left(\xi_{1}+\frac{\lambda_{l}^{2}}{\lambda_{c}^{2}}\right)\frac{\partial {\tilde{M}}^{NStressG}\left(0,t\right)}{\partial \tilde{x}}$$

(A6) $$-\frac{\partial^{3}\tilde{w}\left(1,t\right)}{\partial {\tilde{x}}^{3}}-\frac{1}{\lambda_{c}}\frac{\partial^{2}\tilde{w}\left(1,t\right)}{\partial {\tilde{x}}^{2}}=\;\;\frac{\xi_{1}}{\lambda_{c}}{\tilde{M}}^{NStressG}\left(1,t\right)+\;\left(\xi_{1}+\frac{\lambda_{l}^{2}}{\lambda_{c}^{2}}\right)\;\frac{\partial {\tilde{M}}^{NStressG}\left(1,t\right)}{\partial \tilde{x}}$$

In addition, the bending moment in dimensionless form can be rewritten as

(A7) $${\tilde{M}}^{NStressG}\left(\tilde{x},t\right)=-\;\frac{\partial^{2}\tilde{w}\left(\tilde{x},t\right)\;}{\partial {\tilde{x}}^{2}}+\lambda_{c}^{2}\frac{\partial^{4}\tilde{w}\left(\tilde{x},t\right)\;}{\partial {\tilde{x}}^{4}}+\tilde{A_{\rho }}\left(\lambda_{c}^{2}\xi_{1}+\lambda_{l}^{2}\right)\left(\frac{\partial^{2}\tilde{w}\left(\tilde{x},t\right)}{\partial t^{2}}-{\tilde{g}}^{2}\frac{\partial^{4}\tilde{w}\left(\tilde{x},t\right)}{\partial {\tilde{x}}^{2}\partial t^{2}}\right)\;\;\;\;\;\;\;\;\;\;\;\;\;\;\;\;\;\;\;\;\;\;\;\;\;\;\;\;\;\;\;\;\;\;\;\;\;\;\;\;\;\;\;\;\;\;\;\;\;\;\;\;\;\;+\left(\lambda_{c}^{2}\;\xi_{1}+\lambda_{l}^{2}\right)\left(\left(\tilde{N^{T}}+\tilde{N^{H}}-\left(\tilde{r^{2}}\int_{0}^{1}\left(\frac{1}{2}{\left(\frac{\partial \tilde{w}\left(\tilde{x},t\right)}{\partial \tilde{x}}\right)}^{2}-\lambda_{c}^{2}\frac{\partial^{3}\tilde{w}\left(\tilde{x},t\right)}{\partial {\tilde{x}}^{3}}\right)dz\right)\right)\frac{\partial^{2}\tilde{w}\left(\tilde{x},t\right)}{\partial {\tilde{x}}^{2}}\right)$$

The linear transverse free vibrations associated with NStressG constitutive formulation are obtained by assuming $\tilde{r^{2}}=0$ in Equation (A2)

- Dimensionless linear free vibration equation
  (A8) $$\lambda_{c}^{2}\frac{\partial^{6}\tilde{w}\left(\tilde{x},t\right)}{\partial {\tilde{x}}^{6}}-\frac{\partial^{4}\tilde{w}\left(\tilde{x},t\right)}{\partial {\tilde{x}}^{4}}+\lambda_{c}^{2}\left(\xi_{1}+\frac{\lambda_{l}^{2}}{\lambda_{c}^{2}}\right)\left(\tilde{N^{T}}+\tilde{N^{H}}\right)\frac{\partial^{4}\tilde{w}\left(\tilde{x},t\right)}{\partial {\tilde{x}}^{4}}-\left(\tilde{N^{T}}+\tilde{N^{H}}\right)\frac{\partial^{2}\tilde{w}\left(\tilde{x},t\right)}{{\tilde{x}}^{2}}\;\;\;\;\;\;\;\;\;\;\;\;\;\;\;\;\;\;\;\;\;\;\;\;\;\;\;\;\;\;\;\;\;\;\;\;\;\;\;\;\;\;\;\;\;\;\;\;\;\;\;\;\;\;\;\;\;\;=\tilde{A_{\rho }}\left(\frac{\partial^{2}\tilde{w}\left(\tilde{x},t\right)}{\partial t^{2}}-\tilde{g^{2}}\frac{\partial^{4}\tilde{w}\left(\tilde{x},t\right)}{\partial {\tilde{x}}^{2}\partial t^{2}}\right)-{\tilde{\omega }}^{2}\lambda_{c}^{2}\left(\xi_{1}+\frac{\lambda_{l}^{2}}{\lambda_{c}^{2}}\right)\left(\frac{\partial^{4}\tilde{w}\left(\tilde{x},t\right)}{\partial {\tilde{x}}^{2}\partial t^{2}}-\tilde{g^{2}}\frac{\partial^{6}\tilde{w}\left(\tilde{x},t\right)}{\partial {\tilde{x}}^{4}\partial t^{2}}\right)$$

- Dimensionless standard boundary conditions
  (A9) $$\tilde{w}\left(\tilde{x},t\right)=\tilde{w^{*}}\;\;\mathrm{or}\;\;\frac{\partial {\tilde{M}}^{NStressG}\left(\tilde{x},t\right)}{\partial \tilde{x}}-\left(\tilde{N^{T}}+\tilde{N^{H}}\right)\frac{\partial \tilde{w}\left(\tilde{x},t\right)}{\partial \tilde{x}}=\tilde{V}$$
  (A10) $$-\frac{\partial \tilde{w}\left(\tilde{x},t\right)}{\partial \tilde{x}}=\frac{\partial {\tilde{w}}^{*}}{\partial \tilde{x}}\;\;\;\;\;\;\;\;\mathrm{or}\;\;\;\;\;\;\;{\tilde{M}}^{NStressG}\left(\tilde{x},t\right)=\tilde{M}$$

- Dimensionless constitutive boundary conditions
  (A11) $$-\frac{\partial^{3}\tilde{w}\left(0,t\right)}{\partial {\tilde{x}}^{3}}+\frac{1}{\lambda_{c}}\frac{\partial^{2}\tilde{w}\left(0,t\right)}{\partial {\tilde{x}}^{2}}=-\frac{\xi_{1}}{\lambda_{c}}{\tilde{M}}^{NStressG}\left(0,t\right)+\left(\xi_{1}+\frac{\lambda_{l}^{2}}{\lambda_{c}^{2}}\right)\frac{\partial {\tilde{M}}^{NStressG}\left(0,t\right)}{\partial \tilde{x}}$$
  (A12) $$-\frac{\partial^{3}\tilde{w}\left(1,t\right)}{\partial {\tilde{x}}^{3}}-\frac{1}{\lambda_{c}}\frac{\partial^{2}\tilde{w}\left(1,t\right)}{\partial {\tilde{x}}^{2}}=\;\;\frac{\xi_{1}}{\lambda_{c}}{\tilde{M}}^{NStressG}\left(1,t\right)+\;\left(\xi_{1}+\frac{\lambda_{l}^{2}}{\lambda_{c}^{2}}\right)\;\frac{\partial {\tilde{M}}^{NStressG}\left(1,t\right)}{\partial \tilde{x}}$$

In addition, the bending moment in dimensionless form can be rewritten as

(A13) $${\tilde{M}}^{NStressG}\left(\tilde{x},t\right)=-\;\frac{\partial^{2}\tilde{w}\left(\tilde{x},t\right)\;}{\partial {\tilde{x}}^{2}}+\lambda_{c}^{2}\frac{\partial^{4}\tilde{w}\left(\tilde{x},t\right)\;}{\partial {\tilde{x}}^{4}}+\tilde{A_{\rho }}\left(\lambda_{c}^{2}\xi_{1}+\lambda_{l}^{2}\right)\left(\frac{\partial^{2}\tilde{w}\left(\tilde{x},t\right)}{\partial t^{2}}-{\tilde{g}}^{2}\frac{\partial^{4}\tilde{w}\left(\tilde{x},t\right)}{\partial {\tilde{x}}^{2}\partial t^{2}}\right)\;+\left(\lambda_{c}^{2}\;\xi_{1}+\lambda_{l}^{2}\right)\left(\left(\tilde{N^{T}}+\tilde{N^{H}}\right)\frac{\partial^{2}\tilde{w}\left(\tilde{x},t\right)}{\partial {\tilde{x}}^{2}}\right)$$

The natural frequencies and mode shapes of flexural vibrations are here evaluated by employing the classical separation of spatial and time variables

(A14) $$\tilde{w}\left(\tilde{x},t\right)=\tilde{W}\left(\tilde{x}\right)e^{i\omega t}$$

where *ω* denotes the natural frequency of flexural vibrations.

By substituting Equation (A14) into Equations (A8)–(A13), the following dimensionless governing equations of the linear transverse free vibrations based on NStressG can be rewritten in terms of the non-dimensional spatial shape $\tilde{W}\left(\tilde{x}\right)$ as

- Dimensionless free vibration equation in terms of spatial shape
  (A15) $$\lambda_{c}^{2}\frac{\partial^{6}\tilde{W}\left(\tilde{x}\right)}{\partial {\tilde{x}}^{6}}+\frac{\partial^{4}\tilde{W}\left(\tilde{x}\right)}{\partial {\tilde{x}}^{4}}\left({\tilde{\omega }}^{2}\;\left(\lambda_{c}^{2}\;\xi_{1}+\lambda_{l}^{2}\right){\tilde{g}}^{2}+\left(\lambda_{c}^{2}\;\xi_{1}+\lambda_{l}^{2}\right)\left(\tilde{N^{T}}+\tilde{N^{H}}\right)-1\right)\;\;\;\;\;\;\;\;\;\;\;\;\;\;\;\;\;-\frac{\partial^{2}\tilde{W}\left(\tilde{x}\right)}{{\tilde{x}}^{2}}\left({\tilde{\omega }}^{2}\left(\lambda_{c}^{2}\xi_{1}+\lambda_{l}^{2}\right)+{\tilde{g}}^{2}{\tilde{\omega }}^{2}+\left(\tilde{N^{T}}+\tilde{N^{H}}\right)\right)+{\tilde{\omega }}^{2}\tilde{W}\left(\tilde{x}\right)=0$$

- Dimensionless standard boundary conditions in terms of spatial shape
  (A16) $$\tilde{W}\left(\tilde{x}\right)=\tilde{W^{*}}\;\;\;\;\;\;\;\;\;\;\mathrm{or}\;\;\;\;\;\;\;\;\;\;\;\frac{\partial {\tilde{M}}^{NStressG}\left(\tilde{x}\right)}{\partial \tilde{x}}-\left(\tilde{N^{T}}+\tilde{N^{H}}\right)\frac{\partial \tilde{W}\left(\tilde{x}\right)}{\partial \tilde{x}}=\tilde{V}$$
  (A17) $$-\frac{\partial \tilde{W}\left(\tilde{x}\right)}{\partial \tilde{x}}=\frac{\partial {\tilde{W}}^{*}}{\partial \tilde{x}}\;\;\;\;\;\;\mathrm{or}\;\;\;\;\;\;\;\;\;\;\;{\tilde{M}}^{NStressG}\left(\tilde{x}\right)=\tilde{M}$$

- Dimensionless constitutive boundary conditions in terms of spatial shape
  (A18) $$-\frac{\partial^{3}\tilde{W}\left(0\right)}{\partial {\tilde{x}}^{3}}+\frac{1}{\lambda_{c}}\frac{\partial^{2}\tilde{W}\left(0\right)}{\partial {\tilde{x}}^{2}}=-\frac{\xi_{1}}{\lambda_{c}}{\tilde{M}}^{NStressG}\left(0\right)+\left(\xi_{1}+\frac{\lambda_{l}^{2}}{\lambda_{c}^{2}}\right)\frac{\partial {\tilde{M}}^{NStressG}\left(0\right)}{\partial \tilde{x}}\;$$
  (A19) $$-\frac{\partial^{3}\tilde{W}\left(1\right)}{\partial {\tilde{x}}^{3}}-\frac{1}{\lambda_{c}}\frac{\partial^{2}\tilde{W}\left(1\right)}{\partial {\tilde{x}}^{2}}=\frac{\xi_{1}}{\lambda_{c}}{\tilde{M}}^{NStressG}\left(1\right)+\left(\xi_{1}+\frac{\lambda_{l}^{2}}{\lambda_{c}^{2}}\right)\frac{\partial {\tilde{M}}^{NStressG}\left(1\right)}{\partial \tilde{x}}\;$$

- Dimensionless bending moment in terms of spatial shape
  (A20) $$\begin{array}{cc} {\tilde{M}}^{NStressG}\left(\tilde{x}\right)= & \lambda_{c}^{2}\frac{\partial^{4}\tilde{W}\left(\tilde{x}\right)\;}{\partial {\tilde{x}}^{4}}\\ & +\frac{\partial^{2}\tilde{W}\left(\tilde{x}\right)\;}{\partial {\tilde{x}}^{2}}\left({\tilde{\omega }}^{2}\left(\lambda_{c}^{2}\xi_{1}+\lambda_{l}^{2}\right){\tilde{g}}^{2}+\left(\lambda_{c}^{2}\xi_{1}+\lambda_{l}^{2}\right)\left(\tilde{N^{T}}+\tilde{N^{H}}\right)-1\right)\\ & -{\tilde{\omega }}^{2}\left(\lambda_{c}^{2}\xi_{1}+\lambda_{l}^{2}\right)\tilde{W}\left(\tilde{x}\right)\; \end{array}$$

The analytical solution of Equation (A15) can be expressed in the following form

(A21) $$\tilde{W}\left(\tilde{x}\right)=\overset{6}{\underset{k=1}{\sum }}q_{k}e^{\tilde{x}\beta_{k}}\;$$

wherein $\beta_{k}\;$ are the roots of the characteristic equation and $q_{k}\;$ are six unknown constants to be determined by imposing suitable boundary conditions.

Note that, the six unknown constants can be obtained by satisfying boundary conditions Equations (A16)–(A19). Lastly, the linear fundamental natural frequencies of an FG nano-beam consists of solving the eigenvalue problem expressed in terms of a six dimensional array, $q=\left\{q_{1},\ldots ,q_{6}\right\}$. It can be noted that the corresponding characteristic equation is strongly nonlinear and is numerically solved by using a Wolfram language code written by the authors in Mathematica.

## References

[1] Thostenson E.T., Ren Z.F., Chou T.W. (2001). Advances in the science and technology of carbon nanotubes and their composite: A review. Compos. Sci. Technol..

[2] Lau K.T., Hui D. (2002). The revolutionary creation of new advanced materials-carbon nanotube composites. Compos. Part B Eng..

[3] Gou J., Minaie B., Wang B., Liang Z., Zhang C. (2004). Computational and experimental study of interfacial bonding of single-walled nanotube reinforced composites stiffness. Comput. Mater. Sci..

[4] Liu F., Hu N., Zhang J.Y., Atobe S., Weng S.Y., Ning H.M., Liu Y.L., Wu L.K., Zhao Y.X., Mo F.H. (2016). The interfacial mechanical properties of functionalized graphene-polymer nanocomposites. RSC Adv..

[5] Fasolino A., Los J.H., Katsnelson M.I. (2007). Intrinsic ripples in graphene. Nat. Mater..

[6] Young R.J., Kinloch I.A., Gong L., Novoselov K.S. (2012). The mechanics of graphene nanocomposites: A review. Compos. Sci. Technol..

[7] Du J.H., Cheng H.M. (2012). The fabrication, properties, and uses of graphene/polymer composites. Macromol. Chem. Phys..

[8] Calvert P. (1999). Nanotube composites—A recipe for strength. Nature.

[9] Herron N., Thorn D.L. (1998). Nanoparticles: Uses and relationships to molecular cluster compounds. Adv. Mater..

[10] Huang P., Shi H.Q., Fu S.Y., Xiao H.M., Hu N., Li Y.Q. (2016). Greatly decreased redshift and largely enhanced refractive index of mono-dispersed ZnO-QD/silicone nanocomposites. J. Mater. Chem..

[11] Fu S., Sun Z., Huang P., Li Y., Hu N. (2019). Some basic aspects of polymer nanocomposites: A critical review. Nano Mater. Sci..

[12] Bhattacharya M. (2016). Polymer Nanocomposites—A Comparison between Carbon Nanotubes, Graphene, and Clay as Nanofillers. Materials.

[13] Moskalyuk O.A., Belashov A.V., Beltukov Y.M., Ivan’kova E.M., Popova E.N., Semenova I.V., Yelokhovsky V.Y., Yudin V.E. (2020). Polystyrene-Based Nanocomposites with Different Fillers: Fabrication and Mechanical Properties. Polymers.

[14] Vodenitcharova T., Zhang L.C. (2006). Bending and local buckling of a nanocomposite beam reinforced by a single walled carbon nanotube. Int. J. Solids. Struct..

[15] Anirudh B., Ben Zineb T., Polit O., Ganapathi M., Prateek G. (2020). Nonlinear bending of porous curved beams reinforced by functionally graded nanocomposite graphene platelets applying an efficient shear flexible finite element approach. Int. J. Nonlinear Mech..

[16] Mayandi K., Jeyaraj P. (2003). Bending, buckling and free vibration characteristics of FG-CNT-reinforced polymer composite beam under non-uniform thermal load. J. Mater. Design Appl..

[17] Shen H.C., Lin F., Xiang Y. (2017). Nonlinear bending and thermal postbuckling of functionally graded graphene-reinforced composite laminated beams resting on elastic foundations. Eng. Struct..

[18] Wang Y., Xie K., Fu T., Shi C. (2019). Bending and elastic vibration of a novel functionally graded polymer nanocomposite beam reinforced by graphene nanoplatelets. Nanomaterials.

[19] Wattanasakulpong N., Ungbhakorn V. (2013). Analytical solutions for bending, buckling and vibration responses of carbon nanotube-reinforced composite beams resting on elastic foundation. Comput. Mater. Sci..

[20] Yas M.H., Samadi N. (2012). Free vibrations and buckling analysis of carbon nanotube-reinforced composite Timoshenko beams on elastic foundation. Int. J. Press. Vessels Pip..

[21] Alibeigloo A. (2016). Elasticity solution of functionally graded carbon nanotube-reinforced composite cylindrical panel subjected to thermo mechanical load. Compos. Part B Eng..

[22] Shen H.S. (2011). Postbuckling of nanotube-reinforced composite cylindrical shells in thermal environments, Part I: Axially-loaded shells. Compos. Struct.

[23] Wang Z.X., Shen H.S. (2011). Nonlinear vibration of nanotube-reinforced composite plates in thermal environments. Comput. Mater. Sci..

[24] Zhu P., Lei Z.X., Liew K.M. (2012). Static and free vibration analyses of carbon nanotubereinforced composite plates using finite element method with first order shear deformation plate theory. Compos. Struct..

[25] Wang Z.X., Shen H.S. (2012). Nonlinear vibration and bending of sandwich plates with nanotube-reinforced composite face sheets. Compos. Part. B Eng..

[26] Borjalilou V., Taati E., Ahmadian M.T. (2019). Bending, buckling and free vibration of nonlocal FG-carbon nanotube-reinforced composite nanobeams: Exact solutions. SN Appl. Sci..

[27] Daikh A.A., Drai A., Houari M.S.A., Eltaher M. (2020). Static analysis of multilayer nonlocal strain gradient nanobeam reinforced by carbon nanotubes. Steel Compos. Struct..

[28] Daikh A.A., Houari M.S.A., Karami B., Eltaher M., Dimitri R., Tornabene F. (2021). Buckling Analysis of CNTRC Curved Sandwich Nanobeams in Thermal Environment. Appl. Sci..

[29] Marotti e Sciarra F., Russo P. (2019). Experimental Characterization, Predictive Mechanical and Thermal Modeling of Nanostructures and Their Polymer Composites.

[30] Eringen A.C. (1972). Linear theory of nonlocal elasticity and dispersion of plane waves. Int. J. Eng. Sci..

[31] Eringen A.C. (1983). On differential equations of nonlocal elasticity and solutions of screw dislocation and surface waves. J. Appl. Phys..

[32] Romano G., Barretta R. (2017). Nonlocal elasticity in nanobeams: The stress-driven integral model. Int. J. Eng. Sci..

[33] Fernández-Sáez J., Zaera R., Loya J.A., Reddy J.N. (2016). Bending of Euler–Bernoulli beams using Eringen’s integral formulation: A paradox resolved. Int. J. Eng. Sci..

[34] Romano G., Barretta R., Diaco M., Marotti de Sciarra F. (2017). Constitutive boundary conditions and paradoxes in nonlocal elastic nano-beams. Int. J. Mech. Sci..

[35] Barretta R., Fazelzadeh S.A., Feo L., Ghavanloo E., Luciano R. (2018). Nonlocal inflected nano-beams: A stress-driven approach of bi-Helmholtz type. Compos. Struct..

[36] Barretta R., Luciano R., Marotti de Sciarra F., Ruta G. (2018). Stress-driven nonlocal integral model for Timoshenko elastic nano-beams. Eur. J. Mech. A Solids.

[37] Barretta R., Canadija M., Feo L., Luciano R., Marotti de Sciarra F., Penna R. (2018). Exact solutions of inflected functionally graded nano-beams in integral elasticity. Compos. Part B Eng..

[38] Diaco M., Feo L., Luciano R., Marotti de Sciarra F., Penna R. (2018). Stress-driven integral elastic theory for torsion of nano-beams. Mech. Res. Commun..

[39] Barretta R., Fabbrocino F., Luciano R., Marotti de Sciarra F. (2018). Closed-form solutions in stress-driven two-phase integral elasticity for bending of functionally graded nano-beams. Phys. E Low-Dimens. Syst. Nanostruct..

[40] Apuzzo A., Barretta R., Luciano R., Marotti de Sciarra F., Penna R. (2017). Free vibrations of Bernoulli–Euler nano-beams by the stress-driven nonlocal integral model. Compos.Part B Eng..

[41] Barretta R., Faghidian S.A., Luciano R., Medaglia C.M., Penna R. (2018). Stress-driven two-phase integral elasticity for torsion of nano-beams. Compos.Part B Eng..

[42] Penna R., Feo L. (2020). Nonlinear Dynamic Behavior of Porous and Imperfect Bernoulli-Euler Functionally Graded Nanobeams Resting on Winkler Elastic Foundation. Technologies.

[43] Darban H., Fabbrocino F., Feo L., Luciano R. (2020). Size-dependent buckling analysis of nanobeams resting on two-parameter elastic foundation through stress-driven nonlocal elasticity model. Mech. Adv. Mater. Struct..

[44] Penna R., Feo L., Fortunato A., Luciano R. (2021). Nonlinear free vibrations analysis of geometrically imperfect FG nano-beams based on stress-driven nonlocal elasticity with initial pretension force. Compos. Struct..

[45] Lim C.W., Zhang G., Reddy J.N. (2015). A higher-order nonlocal elasticity and strain gradient theory and its applications in wave propagation. J. Mech. Phys. Solids.

[46] Zaera R., Serrano Ó., Fernández-Sáez R. (2019). On the consistency of the nonlocal strain gradient elasticity. Int. J. Eng. Sci..

[47] Eringen A.C. (1984). Theory of Nonlocal Elasticity and Some Applications. Princet. Univ. Nj Dept. Civ. Eng..

[48] Fernández-Sáez J., Zaera R. (2017). Vibrations of Bernoulli–Euler beams using the two-phase nonlocal elasticity theory. Int. J. Eng. Sci..

[49] Khaniki H.B. (2018). Vibration analysis of rotating nanobeam systems using Eringen’s two-phase local/nonlocal model. Phys. ELow-Dimens. Syst. Nanostruct..

[50] Gurtin M.E., Murdoch A.I. (1975). A continuum theory of elastic material surfaces. Arch. Ration. Mech. Anal..

[51] Zhu X., Li L. (2019). A well-posed Euler-Bernoulli beam model incorporating nonlocality and surface energy effect. Appl. Math. Mech..

[52] Barretta R., Marotti de Sciarra F. (2019). Variational nonlocal gradient elasticity for nano-beams. Int. J. Eng. Sci..

[53] Pinnola F.P., Faghidian S.A., Barretta R., Marotti de Sciarra F. (2020). Variationally consistent dynamics of nonlocal gradient elastic beams. Int. J. Eng. Sci..

[54] Penna R., Lovisi G., Feo L. (2021). Hygro-thermal bending behavior of porous FG nano-beams via local/nonlocal strain and stress gradient theories of elasticity. Compos. Struct..

[55] Penna R., Feo L., Lovisi G., Fabbrocino F. (2021). Hygro-Thermal Vibrations of Porous FG Nano-Beams Based on Local/Nonlocal Stress Gradient Theory of Elasticity. Nanomaterials.

